# Chemical signatures and sensory perception of *Nongxiangxing Baijiu*: regional and quality-grade discrimination and the modulatory role of ethanol

**DOI:** 10.1016/j.fochx.2026.103790

**Published:** 2026-03-26

**Authors:** Ruotao Zhou, Ran Xiangli, Dong Zhao, Jia Zheng, Jian Su, Zheng Feng, Yue Ma, Yan Xu

**Affiliations:** a Laboratory of Brewing Microbiology and Applied Enzymology, School of Biotechnology and Key Laboratory of Industrial Biotechnology of Ministry of Education, Jiangnan University, Wuxi, Jiangsu, PR China; b China Key Laboratory of Microbiomics and Eco-Brewing Technology for Light Industry, Wuxi, Jiangsu, PR China; c Sichuan Key Laboratory of Solid-State Fermentation Resources Utilization; Wuliangye Yibin Co., Ltd, Yibin, Sichuan 644000, PR China

**Keywords:** *Nongxiangxing base Baijiu*, Quality grades, Regions, Sensory evaluation, Odorants, Alcohol reduction

## Abstract

This study systematically characterized the sensory and chemical features of five-grain *Nongxiangxing base Baijiu* across production regions and quality grades. The results showed differences in both overall sensory profiles and chemical composition between samples from the Yibin region and those from other regions. Partial least squares discriminant analysis (PLS-DA) differentiated Yibin from Non-Yibin samples and identified 29 candidate discriminant compounds. Between quality grades, compound composition also differed. Mann–Whitney *U* tests further identified 34 compounds with significant differences between grades, with esters and alcohols enriched in premium-quality grade samples and acids higher in normal-quality grade samples. Rate-All-That-Apply (RATA) results indicated that ethanol reduction via water dilution altered sensory profiles: although aroma-compound concentrations decreased proportionally with dilution, sensory profiles did not decrease proportionally. Overall, this study further clarifies regional and quality-grade differentiation in five-grain *Nongxiangxing base Baijiu* and highlights the important role of ethanol in aroma perception.

## Highlights

• Yibin and non-Yibin *Nongxiangxing base Baijiu* differed in overall sensory profiles.
• PLS-DA identified 29 candidate compounds distinguishing Yibin/non-Yibin samples.
• Mann-Whitney *U* tests identified 34 compounds with significant grade differences.
• Ethanol reduction via water dilution altered sensory profiles of the samples.

## 1 Introduction

Baijiu is a traditional Chinese distilled spirit with a long history and significant cultural and economic value in Chinese society. Due to variations in raw materials, production techniques, and environmental conditions (Wei et al., 2020), Baijiu has evolved into a diverse system of flavor styles, with 12 major aroma types being widely recognized (Xu et al., 2022). Among them, *Nongxiangxing Baijiu*, one of the four fundamental aroma types, is highly favored by consumers for its rich taste and well-balanced aroma profile. In 2023, the sales of *Nongxiangxing Baijiu* accounted for approximately 50% of the overall Chinese Baijiu market (Ou, 2025).

In the Baijiu industry, catering to consumer flavor preferences is central to product value. Commercial Baijiu is typically produced by blending different styles of base Baijiu in specific proportions, making the flavor characteristics of *Nongxiangxing base Baijiu* critical determinants of the final product's aroma and quality. Therefore, there is a need to establish a systematic research framework to investigate *Nongxiangxing base Baijiu*, comprehensively characterize its sensory attributes and chemical basis, and compare style-specific differences. Such efforts can improve blending efficiency, enhance the flavor quality of commercial Baijiu, and promote long-term stability in product quality.

Based on raw material formulations, *Nongxiangxing Baijiu* is generally classified into multi-grain (sorghum, rice, glutinous rice, wheat, and corn) and single-grain (predominantly sorghum) types (Chen et al., 2026), and is mainly produced in three major regions: Sichuan, Jianghuai, and northern China (Hong et al., 2023). Among them, Yibin (Sichuan) serves as the core production area for five-grain *Nongxiangxing Baijiu* and has given rise to iconic brands such as Wuliangye. Yibin has a humid subtropical monsoon climate, with an average annual temperature of approximately 18 °C, high humidity, limited sunshine, and low wind speed. Together with abundant water resources and mineral availability, this environment provides favorable conditions for the enrichment, growth, and metabolism of brewing microorganisms. In addition, the humus content in pit mud from Yibin is significantly higher than that in other regions. As a colloidal substance formed through the microbial decomposition and metabolism of organic matter, humus provides a favorable environment for microbial growth, improves the physicochemical properties of pit mud, and may contribute to Baijiu quality (Ning et al., 2025). Furthermore, aged pit mud from Yibin is enriched in dominant brewing microorganisms. For example, JNU-WLY1368 has been reported to be highly enriched in Yibin pit mud as a key caproate-producing bacterium, and it exhibits one of the broadest known substrate-utilization spectra (Wang et al., 2021). The enrichment of such flavor-relevant microorganisms provides a crucial biological basis for the rich aroma profile of Yibin *Nongxiangxing Baijiu*. From a process-technology perspective, both the Daqu-making temperature and the fermentation temperature in Yibin are typically higher than those in other regions, which may promote the Maillard reaction, thereby increasing the formation of pyrazines and furans (Song et al., 2020).

Although previous studies have compared flavor-chemical differences among *Nongxiangxing Baijiu* from different regions (He et al., 2020; Song et al., 2020; Wang et al., 2025), Sichuan is often treated as a single production region without distinguishing sub-regions such as Luzhou and Yibin. This simplification is nontrivial because the two sub-regions differ markedly in raw material formulations (Luzhou largely uses a single-grain formulation, whereas Yibin is dominated by five-grain formulations). In addition, the flavor chemistry and sensory characterization of *Nongxiangxing base Baijiu* remain relatively underexplored. As *base Baijiu* has not yet been blended, its chemical composition and sensory properties can more directly reflect regional style. Therefore, a systematic comparison of five-grain *Nongxiangxing base Baijiu* from Yibin and those from other regions would help define Yibin's regional style more clearly and provide a stronger basis for region-specific differentiation.

Blending is one of the most critical steps in Baijiu production, determining the final style and quality of commercial Baijiu. Before blending, *Nongxiangxing base Baijiu* is graded and classified through a complex process performed by experienced tasters based on sensory attributes (Gong et al., 2023). However, sensory evaluation is prone to bias, leading to inconsistent grading, which may compromise blending precision and the quality stability of commercial Baijiu. Establishing an objective, material-based classification system for *Nongxiangxing base Baijiu* is of profound significance for advancing standardized production in the Baijiu industry. In recent years, studies have attempted to develop material-based models for grading the quality of base Baijiu (Li et al., 2025; Zhou et al., 2025). However, most existing work has focused on a single brand or a single production area, where sample origins and production processes are relatively homogeneous. Whether the grading criteria for base Baijiu share commonalities across different production regions, and whether a relatively stable set of marker compounds exists that can discriminate quality grades across regions, remain insufficiently explored.

The alcohol content of *Nongxiangxing base Baijiu* is typically high (usually exceeding 60% vol), which may cause oral and gastrointestinal irritation during consumption, making ethanol reduction an unavoidable step in the blending process. Additionally, to cater to younger consumer markets, the development of lower-alcohol Baijiu products has become an inevitable trend. Ethanol and water together account for more than 98% of Baijiu's composition and serve as the solvent matrix for thousands of aroma compounds (Duan et al., 2022). Changes in ethanol concentration can therefore systematically affect the solubility, odor thresholds, and volatility of aroma compounds, ultimately altering the overall aroma profile. Across alcoholic beverage systems, ethanol content has been shown to influence aroma perception: in *Jiangxiangxing Baijiu*, high- versus low-alcohol products differ in aroma, with low-alcohol products more prone to oxidized oil-like off-odors (Zhang, Lu, Yu, Chen and Xu, 2025a; Zhang, Yu, Chen and Xu, 2025b); in beer, increased ethanol reduces the perception of off-flavors (Perpète & Collin, 2000); and in *Qingxiangxing Baijiu*, the unpleasant musty/bran-like odor increases significantly during dilution (Lu et al., 2021). However, little is known about the effects of ethanol reduction on the aroma profile of *Nongxiangxing base Baijiu*. Clarifying whether, and how, ethanol reduction affects aroma perception is critical for optimizing alcohol-reduction strategies while maintaining product style, balance, and drinking quality.

Based on the above, this study analyzed five-grain *Nongxiangxing base Baijiu* from different production regions and quality grades, focusing on: (1) Characterizing the sensory profiles and compound composition differences of *Nongxiangxing base Baijiu* from Yibin and those from other regions (hereafter referred to as the Non-Yibin group). (2) Characterizing the chemical differences between premium-quality grade and normal-quality grade *Nongxiangxing base Baijiu* across the sampled regions, and screening candidate marker compounds associated with quality classification. (3) Characterizing changes in the sensory profile of *Nongxiangxing base Baijiu* after water dilution for ethanol reduction.

## 2 Materials and methods

### 2.1 *Nongxiangxing base Baijiu* samples

The 14 *Nongxiangxing base Baijiu* samples used in this study were all produced using a five-grain formulation and were provided by 7 Baijiu manufacturers from 4 different *Nongxiangxing Baijiu* production areas. Each manufacturer supplied samples representing two quality grades: premium-quality grade and normal-quality grade. All samples were evaluated by experts from the China Alcoholic Drinks Association to confirm that they were representative of their respective production regions. The samples were stored in brown glass bottles, the headspace was flushed with high-purity nitrogen, tightly sealed, and maintained under constant-temperature conditions. Detailed information on the samples is provided in Table 1.

**Table 1:** Information of *Nongxiangxing base Baijiu* samples.

No.	Sample code	Region	Quality grade	Alcohol content (% vol)
1	A-G1-G	Anhui	premium	69.8
2	G-J1-G	Gansu	premium	72.7
3	J-Y1-G	Jiangsu	premium	53.3
4	S-Y1-G	Sichuan	premium	71.0
5	S-Y2-G	Sichuan	premium	70.0
6	S-Y3-G	Sichuan	premium	68.5
7	S-Y4-G	Sichuan	premium	73.5
8	A-G1-Z	Anhui	normal	68.0
9	G-J1-Z	Gansu	normal	69.3
10	J-Y1-Z	Jiangsu	normal	68.3
11	S-Y1-Z	Sichuan	normal	67.3
12	S-Y2-Z	Sichuan	normal	68.9
13	S-Y3-Z	Sichuan	normal	68.0
14	S-Y4-Z	Sichuan	normal	68.0

### 2.2 Reagents and chemicals

High-purity solvents (≥99.8%, HPLC grade), including absolute ethanol, dichloromethane, ethyl acetate, and acetone, as well as chromatographic-grade chemical standards (purity ≥97%) and internal standards (ISs) used for quantification (Table A.1) were purchased from Sigma-Aldrich Trading Co., Ltd. (Shanghai, China). Anhydrous sodium sulfate (Na₂SO₄, analytical grade) and sodium chloride (NaCl, analytical grade) were obtained from China National Pharmaceutical Group Corp. (Shanghai, China). Ultrapure water was produced using a Milli-Q purification system (Millipore, Bedford, MA, USA).

### 2.3 Sensory analysis

All sensory experiments in this study were conducted in full compliance with the Declaration of Helsinki and were approved by the Ethics Committee of Jiangnan University (JNU20220901IRB20). All participants were fully informed about the experimental procedures and provided written informed consent prior to participation. Panelists received appropriate compensation or small gifts as appreciation for their involvement.

According to the Chinese national standard GB/T 33404–2016, Baijiu samples were opened 15 min prior to each sensory session, and 8 mL of each sample was poured into standard Baijiu tasting glasses. Each sample was labeled with a randomly generated three-digit code and presented to panelists in a randomized order to minimize potential order effects. The sensory room temperature was maintained at 23 °C throughout the experiments. Participants were instructed to refrain from eating or drinking for at least 1 h before testing and to avoid using any scented products on the day of the experiment.

#### 2.3.1 Rate-all-that-apply (RATA)

The recruitment, screening, and training of panelists, as well as the specific procedures for sensory evaluation, were conducted according to the methods described in a previous study (Xiangli et al., 2024). In brief, 21 participants (11 females and 10 males, aged 19–25 years) were recruited from Jiangnan University to form the sensory panel. All panelists successfully completed the sensory screening and training sessions and demonstrated good ability to discriminate between Baijiu samples and evaluate flavor attributes.

During the formal experiment, 14 *Nongxiangxing base Baijiu* samples were each diluted to 52% vol and 42% vol, resulting in a total of 28 samples and their sensory attributes were assessed using the Rate-All-That-Apply (RATA) method. A total of 18 sensory attributes were selected through preliminary discussions, including 11 aroma attributes (apple, pineapple, sorghum, qu, alcohol, honey, mushroom, grass, animal & sweaty, earth, leather) and 7 taste and mouthfeel attributes (sour, sweet, bitter, pungent, astringent, refreshing, soft). The definitions and reference standards for these attributes have been detailed in our previous publication (Xiangli et al., 2024).

### 2.4 Quantitative analysis of odor-active compounds using multiple analytical methods

A total of 96 odor-active compounds, identified based on our previous research (Ma et al., 2025), were selected as target analytes for quantitative analysis, following the quantification methods described in that study. To ensure consistency in quantification across samples, the ethanol content of all Baijiu samples was adjusted to 52% vol prior to analysis. Quantification was performed using calibration curves with internal standard correction, and the calibration curves for all target compounds were identical to those used in the previously published study.

#### 2.4.1 Direct injection (DI) gas chromatography-flame ionization detection (GC-FID) analysis

GC-FID with direct injection was used to quantify several high-concentration compounds. For each analysis, 1 mL of sample was spiked with 50 μL of an internal standard solution containing IS1 (2-methylbutan-2-ol, 2.33 mg/L) and IS2 (pentyl acetate, 2.64 mg/L), and a 1 μL aliquot was injected into the GC-FID. The GC-FID system, chromatographic column, oven temperature program, split ratio, carrier gas conditions, and injector and detector temperatures employed for quantification were identical to those reported in our previous study.

All analyses were performed in triplicate, and mean values were reported.

#### 2.4.2 Liquid-liquid microextraction (LLME) combined with GC–MS

Several low-abundance compounds, including volatile organic acids, heterocyclic compounds, selected alcohols, and phenols, were quantified using liquid-liquid microextraction coupled with GC–MS (LLME-GC–MS). The internal standards used are as follows: IS3: 3,3-dimethylbutanoic acid (100 μL, 200.02 mg/L), IS4: isotopically labeled guaiacol-d₃ (40 μL, 49.99 mg/L), IS5: furfural-d₄ (20 μL, 237.50 mg/L), IS6: 2-methoxy-3-(2-methylpropyl)pyrazine (20 μL, 200.06 mg/L), and IS7: isotopically labeled hexan-1-ol-d₁₃ (24.6 μL, 162.70 mg/L).

Each sample was diluted with ultrapure water to a final volume of 20 mL, adjusting the alcohol content to 10% vol. Internal standards were then added, and the solution was saturated with sodium chloride. The extractant (ethyl acetate) and dispersant (acetone) were premixed at a 1:1 ratio, and 4 mL of the mixture was added to each sample. Extraction was carried out on a shaker for 10 min. After phase separation, the upper organic layer was collected and concentrated to 250 μL under a gentle stream of nitrogen. A 1 μL aliquot of the concentrated extract was injected into the GC–MS for analysis. The GC–MS operating conditions were identical to those reported in our previous study.

#### 2.4.3 Headspace solid-phase microextraction followed by GC–MS (HS-SPME-GC–MS)

Quantification of the remaining compounds was carried out using GC–MS equipped with an automated headspace SPME Arrow injector. The internal standards used were IS8: isotopically labeled ethyl octanoate-d₁₅ (5 μL, 988.05 mg/L), IS9: octanal-d₁₆ (5 μL, 500.02 mg/L), IS10: phenylethyl acetate-d₃ (7.1 μL, 247.01 mg/L), IS11: acetophenone-d₃ (7.1 μL, 247.50 mg/L), and IS12: isopropyl disulfide (5 μL, 12.96 mg/L).

All samples were diluted with ultrapure water to a final alcohol content of 10% vol. A 5 mL aliquot of each diluted sample was transferred into a 20 mL brown headspace vial and sealed with a silicone septum. Internal standards were added, followed by the addition of 1.5 g of sodium chloride to saturate the solution. The headspace adsorption and desorption procedures, as well as the GC–MS operating conditions, were identical to our previous study.

### 2.5 Data analysis

The processing of RATA sensory data followed the methods described in a previous study (Xiangli et al., 2024). Sensory data and compound quantification data were initially organized and summarized using Microsoft® Excel® 2021. The following statistical analyses and visualizations were performed in RStudio (version 2024.12.1; Posit Software, PBC, Boston, MA, USA). A PLS-DA model for regional discrimination was constructed based on quantitative volatile-compound data, and score plots were generated (mixOmics, ggplot2, ggrepel). For sensory data, PERMDISP and PERMANOVA were conducted based on Euclidean distance matrices to evaluate differences in the overall sensory profiles between groups (Yibin vs. Non-Yibin), and PCoA plots were generated (vegan, ape, ggplot2). For between-group comparisons (Yibin vs. Non-Yibin) of individual sensory attributes, Mann–Whitney *U* tests were applied with Benjamini–Hochberg FDR correction, and both *p*-values and Cliff's δ were reported (stats, rstatix, effsize). In addition, hierarchical clustering dendrograms and heatmaps were generated (ComplexHeatmap, circlize, dendextend). Python (version 3.12) was used to perform Mann–Whitney U tests to identify compounds showing significant concentration differences between premium-quality grade and normal-quality grade samples. In addition, Wilcoxon signed-rank tests were applied to 18 sensory attributes to evaluate the effects of dilution (52% vol vs. 42% vol) on sensory perception. The main Python libraries used included numpy, pandas, scipy, and matplotlib. Sensory radar plots were generated using OriginPro 2023 (OriginLab Corporation, Northampton, MA, USA).

Statistical significance was set at *p* < 0.05, with the following significance levels: significant (*p* < 0.05, *); highly significant (*p* < 0.01, **); and extremely significant (*p* < 0.001, ***).

## 3 Results and discussion

### 3.1 Differences in sensory profiles between *Nongxiangxing base Baijiu* from the Yibin and non-Yibin production regions

**Fig. 1:**
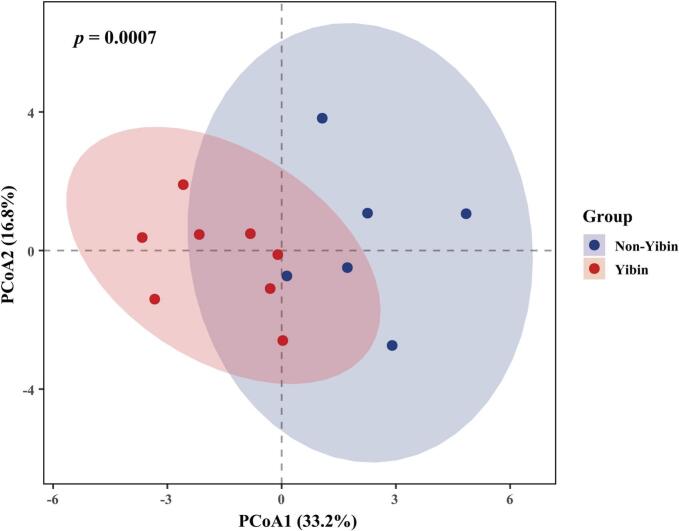
Principal coordinate analysis (PCoA) score plot based on RATA data for *Nongxiangxing base Baijiu* samples from the Yibin and Non-Yibin production regions (Euclidean distance). Ellipses represent 95% confidence intervals. PERMANOVA indicated a significant difference in the overall sensory profiles between samples from the Yibin and Non-Yibin production regions (9999 permutations: *R*^*2*^ = 0.228, *p* = 0.0007).

To examine whether *Nongxiangxing base Baijiu* differs at the sensory level between the Yibin and Non-Yibin regions, all 14 samples were diluted to 52% vol and profiled using Rate-All-That-Apply (RATA). A total of 21 trained panelists rated 18 sensory attributes for each sample. Subsequently, permutational multivariate analysis of variance (PERMANOVA) based on Euclidean distances was employed to determine whether the overall sensory profiles differed between the two groups. The results revealed a significant difference between the Yibin and Non-Yibin groups (*p* = 0.0007), with the grouping factor explaining 22.8% of the between-group variation (*R*^*2*^ = 0.228). To ensure that the PERMANOVA result was not driven by differences in within-group dispersion, permutational analysis of multivariate dispersions (PERMDISP) was conducted using the same distance metric. No significant difference in dispersion was observed between Yibin and the Non-Yibin group (*p* = 0.3513), indicating that the PERMANOVA significance primarily reflects a difference in group centroids rather than unequal within-group variability. Consistently, the Euclidean-distance-based PCoA plot (Fig. 1) showed partial overlap but an overall tendency toward separation between Yibin and Non-Yibin samples.

Subsequently, univariate Mann–Whitney *U* tests were performed to assess between-group differences for each sensory attribute, and *p*-values were adjusted using the Benjamini-Hochberg false discovery rate (BH-FDR) procedure to account for multiple testing. After stringent BH-FDR correction, none of the individual sensory attributes reached statistical significance. However, given that *p*-values are highly sensitive to sample size, we consider that the current sample size may have limited the statistical power to detect between-group differences at the single-attribute level (Park et al., 2026). Since PERMANOVA had already revealed a significant difference in the overall sensory profiles between the Yibin and Non-Yibin groups, Cliff's δ effect size (Cliff, 1993) was used as a complementary metric to evaluate the magnitude and direction of the between-group differences. Applying a threshold of |Cliff's δ| ≥ 0.47 (representing a large effect) (Meissel & Yao, 2024), 10 potentially differential sensory attributes were identified (Table 2), suggesting potential differences between Yibin and Non-Yibin samples.

**Table 2:** Mann–Whitney U test (BH–FDR) and Cliff's δ for sensory attributes comparing Yibin vs Non-Yibin groups.

No.	Attribute	*p*-value^α^	*q*-valueβ	Cliff's δγ	Effect sizeε
1	Animal & sweaty	0.0081	0.1187	−0.875	Large
2	apple	0.014	0.1187	0.813	Large
3	earth	0.0239	0.1187	−0.75	Large
4	bitter	0.033	0.1187	−0.708	Large
5	refreshing	0.033	0.1187	0.708	Large
6	qu	0.0525	0.1576	−0.646	Large
7	pineapple	0.0704	0.181	0.604	Large
8	mushroom	0.1066	0.2132	−0.542	Large
9	alcohol	0.1054	0.2132	0.542	Large
10	leather	0.1547	0.2784	−0.479	Large
11	astringent	0.1962	0.3211	−0.438	Medium
12	sorghum	0.2442	0.3382	−0.396	Medium
13	honey	0.2442	0.3382	0.396	Medium
14	grass	0.3001	0.3858	0.354	Medium
15	sour	0.5613	0.6735	−0.208	Small
16	sweet	0.7466	0.7908	0.125	Negligible
17	soft	0.7469	0.7908	0.125	Negligible
18	pungent	0.9485	0.9485	−0.042	Negligible

**Note:**^α^ The *p*-values were determined using the Mann**–**Whitney U test.

β The *q*-values are *p*-values adjusted for multiple testing using FDR control.

γ A positive Cliff's δ indicates that attribute scores tend to be higher in Yibin samples than in the Non-Yibin group, whereas a negative Cliff's δ indicates that scores tend to be lower in Yibin samples.

ε Effect size interpretation for Cliff's δ is as follows: negligible, |Cliff's δ| < 0.15; small, 0.15 ≤ |Cliff's δ| < 0.33; medium, 0.33 ≤ |Cliff's δ| < 0.47; large, |Cliff's δ| ≥ 0.47 (Meissel & Yao, 2024).

Among the 10 sensory attributes, samples from the Yibin region tended to score higher in “apple,” “refreshing,” “pineapple,” and “alcohol” compared to the Non-Yibin samples, while scoring lower in “animal & sweaty,” “earth,” “bitter,” “qu,” “mushroom,” and “leather.” Overall, the Yibin samples tended to be characterized by fruity and relatively fresh sensory features, while the Non-Yibin samples were more associated with heavier flavor descriptors. It is worth noting that the robustness and statistical significance of these potentially differential attributes still require further validation in a larger sample cohort.

Previous studies have suggested that the sensory characteristics of *Nongxiangxing base Baijiu* are closely related to raw material formulation and region-associated factors (He et al., 2020). In the present study, although all samples were produced using a five-grain formulation to minimize raw-material variation, the Yibin and Non-Yibin samples still exhibited discernible differences in sensory style. These results suggest that, even under relatively consistent raw-material conditions, region-associated factors (such as the local geo-ecological conditions and process-related details) may play an important role in shaping the sensory characteristics of the samples.

### 3.2 Differences in aroma-active compounds between *Nongxiangxing base Baijiu* from the Yibin and non-Yibin production regions

A total of 96 aroma compounds were quantified in this study (see Table A.3). To screen for candidate discriminant compounds that could differentiate the Yibin and Non-Yibin samples, partial least squares discriminant analysis (PLS-DA) was conducted using compound concentrations as explanatory variables (X) and production regions as the response variable (Y). Feature selection was based on variable importance in projection (VIP) scores.

**Fig. 2:**
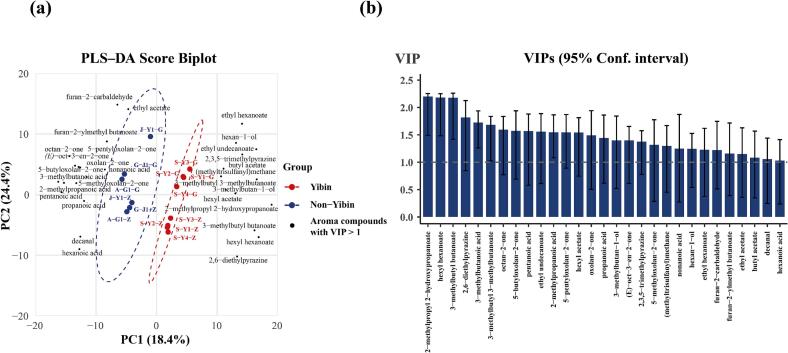
PLS-DA results identifying candidate discriminant compounds between Yibin and Non-Yibin *Nongxiangxing base Baijiu* samples. (a) PLS-DA score-loading biplot showing the separation of Yibin and Non-Yibin samples based on the first two PLS components, along with the loadings of key aroma compounds. (b) Variable importance in projection (VIP) plot highlighting the candidate discriminant compounds (VIP > 1) that contribute most to the differentiation between Yibin and Non-Yibin *Nongxiangxing base Baijiu* samples.

The PLS-DA score plot of the first two components, which explained 42.8% of the variance in X, showed a clear separation between the Yibin and Non-Yibin samples (Fig. 2a). The model was validated using 6-fold cross-validation repeated 500 times, yielding *R*^*2*^*X* = 0.633, R^2^Y = 0.999, and *Q*^*2*^ = 0.685, indicating good discrimination and acceptable predictive performance under internal cross-validation.

Based on VIP > 1, a total of 29 candidate discriminant compounds were identified (Fig. 2b), including 9 esters, 6 acids, 2 alcohols, 3 aldehydes/ketones, 4 lactones, 1 sulfur compound, 2 furans, and 2 pyrazines. According to the loading directions in the score biplot (Fig. 2a), Yibin samples showed negative associations with acids, aldehydes/ketones, lactones, and furans (lower relative concentrations compared with Non-Yibin samples), but positive associations with alcohols and pyrazines. Esters were generally positively associated with Yibin samples, except for ethyl acetate.

The enrichment of esters and pyrazines in Yibin-region *Nongxiangxing base Baijiu* is consistent with previous findings (He et al., 2020; Song et al., 2020). Pyrazines are primarily generated through Maillard reactions, which are promoted under high-temperature conditions (Fan et al., 2007). Since fermentation temperatures in the Yibin region are typically higher than those in the other production regions examined in this study, the elevated pyrazine levels in Yibin samples may be attributed to these process-related factors.

### 3.3 Differences in aroma-active compounds between *Nongxiangxing base Baijiu* of different quality grades

**Fig. 3:**
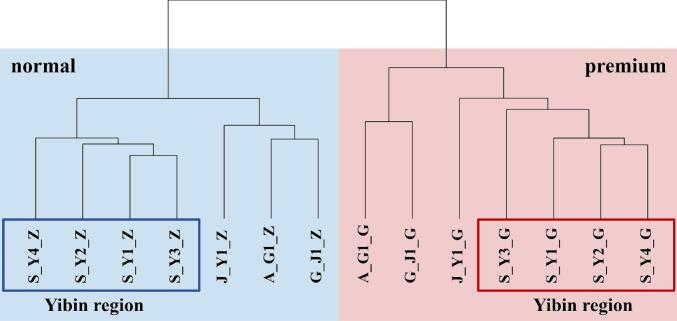
Hierarchical clustering of 14 *Nongxiangxing base Baijiu* samples (52% vol) based on aroma compound concentrations.

Hierarchical cluster analysis (HCA) based on aroma-compound composition clearly separated the premium- and normal-quality grade samples into two distinct clusters; within each grade-specific cluster, Yibin samples showed a separation trend from Non-Yibin samples (Fig. 3). This result suggests that, despite variations in production environments and techniques across regions and manufacturers, samples of the same quality grade exhibit relatively high internal consistency in their aroma-compound composition. Notably, samples of different quality grades from the same manufacturer clustered far apart, indicating substantial differences in compound composition between grades even under identical production conditions. These findings suggest that, within the sampled regions, quality grading of *Nongxiangxing base Baijiu* shows consistent discriminative patterns based on aroma composition.

**Fig. 4:**
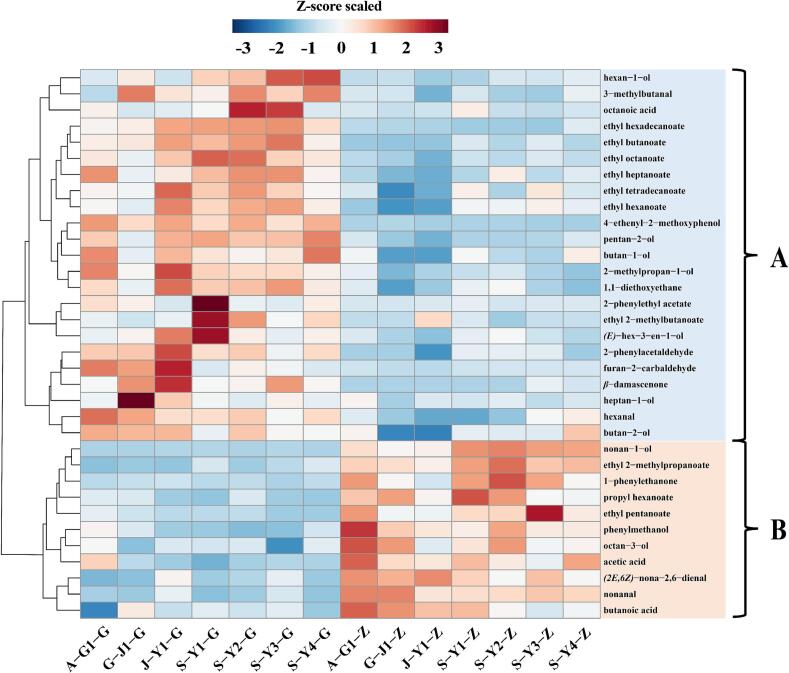
Heatmap and hierarchical clustering analysis of 34 differential aroma compounds (*q* < 0.05) between premium-quality grade and normal-quality grade *Nongxiangxing base Baijiu* samples.

To identify compounds that significantly differed in concentration between quality grades, the Mann–Whitney *U* test was applied to each of the 96 quantified compounds, followed by Benjamini-Hochberg correction to control the false discovery rate (FDR). A total of 34 compounds showed significant differences (*q* < 0.05) between premium- and normal-quality grade samples (Table 3). A hierarchical clustering heatmap was generated to visualize their distribution patterns across grades (Fig. 4).

**Table 3:** Aroma compounds with significantly different concentrations (*q* < 0.05) between premium- and normal-quality grade *Nongxiangxing base Baijiu* samples, along with their odor thresholds and OAVs.

No.	Compound	Threshold (μg/L)	Higher concentration in	*q*-value^α^	Significanceβ	OAV_max_γ
1	ethyl 2-methylpropanoate	57.5 a	normal	0.0006	***	>100
2	ethyl butanoate	81.5 a	premium	0.0006	***	>100
3	2-methylpropan-1-ol	28,300 e	premium	0.0006	***	>1
4	pentan-2-ol	194,313 a	premium	0.0006	***	
5	ethyl pentanoate	26.8 a	normal	0.0006	***	>100
6	propyl hexanoate	12,784 a	normal	0.0006	***	
7	nonanal	122 a	normal	0.0006	***	>10
8	ethyl octanoate	12.9 a	premium	0.0006	***	>100
9	furan-2-carbaldehyde	44,000 a	premium	0.0006	***	
10	2-phenylacetaldehyde	262 d	premium	0.0006	***	>10
11	1-phenylethanone	256 a	normal	0.0006	***	>1
12	nonan-1-ol	806 d	normal	0.0006	***	
13	phenylmethanol	40,927 a	normal	0.0006	***	
14	ethyl hexadecanoate	2000 a	premium	0.0006	***	
15	hexanal	25.5 a	premium	0.0012	**	>100
16	*β*-damascenone	0.12 c	premium	0.0017	**	>100
17	4-ethenyl-2-methoxyphenol	209 a	premium	0.0021	**	
18	1,1-diethoxyethane	2090 a	premium	0.0023	**	>10
19	ethyl heptanoate	13,153 a	premium	0.0023	**	>1
20	*(E)*-hex-3-en-1-ol	400 h	premium	0.0023	**	>1
21	*(2E,6Z)*-nona-2,6-dienal	0.64 b	normal	0.0023	**	>100
22	octan-3-ol	483 a	normal	0.0041	**	
23	3-methylbutanal	16.5 a	premium	0.007	**	>100
24	butan-1-ol	2733 a	premium	0.007	**	>1
25	hexan-1-ol	5370 d	premium	0.007	**	>1
26	acetic acid	160,000 e	normal	0.007	**	>1
27	ethyl 2-methylbutanoate	18.0 a	premium	0.0111	*	>100
28	ethyl hexanoate	55.3 a	premium	0.0111	*	>100
29	heptan-1-ol	26,600 f	premium	0.0111	*	
30	butanoic acid	965 a	normal	0.0111	*	>10
31	butan-2-ol	50,000 g	premium	0.0175	*	
32	2-phenylethyl acetate	909 a	premium	0.0175	*	
33	ethyl tetradecanoate	33,551 b	premium	0.0175	*	
34	octanoic acid	2700 a	premium	0.0175	*	

Note: ^α^ Significance was determined using the Mann–Whitney *U* test, with *p*-values adjusted by false discovery rate (FDR) correction.

β Adjusted *p*-values (*q*-values) were used for significance labeling: “*”, *q* < 0.05; “**”, *q* < 0.01; “***”, *q* < 0.001.

γ The maximum odor activity value (OAV) of each compound across all samples is reported.

a from reference (W. Fan & Xu, 2011).

b from reference (Ma et al., 2025).

c from reference (Pineau et al., 2007).

d from reference (Feng et al., 2025).

e from reference (Gao et al., 2014).

f from reference (H. Fan et al., 2015).

g from reference (Peinado et al., 2004).

h from reference (Zhao & Zheng, 2019).

As shown in Fig. 4, concentrations of Group A compounds were significantly higher in premium-quality grade samples. This group included 23 compounds: 7 esters (ethyl butanoate, ethyl octanoate, ethyl hexadecanoate, ethyl heptanoate, ethyl 2-methylbutanoate, ethyl hexanoate, ethyl tetradecanoate), 7 alcohols (2-methylpropan-1-ol, pentan-2-ol, *(E)*-hex-3-en-1-ol, butan-1-ol, hexan-1-ol, heptan-1-ol, butan-2-ol), 3 aldehydes (hexanal, 1,1-diethoxyethane, 3-methylbutanal), 2 aromatic compounds (2-phenylacetaldehyde, 2-phenylethyl acetate), 1 phenolic compound (4-ethenyl-2-methoxyphenol), 1 acid (octanoic acid), 1 furan derivative (furan-2-carbaldehyde), and 1 terpenoid (*β*-damascenone).

Esters represent one of the most important classes of aroma compounds in Chinese Baijiu. Typically derived from microbial metabolism, they contribute fruity and floral characteristics to the overall aroma profile and are characterized by high abundance, structural diversity, low odor thresholds, and high odor activity values (OAVs) (Mou et al., 2025). In this study, several esters in Group A, such as ethyl butanoate, ethyl octanoate, ethyl 2-methylbutanoate, and ethyl hexanoate, exhibited OAVs >100, indicating exceptionally high aroma activity. Ethyl hexanoate has already been recognized as a key aroma compound in *Nongxiangxing Baijiu* (Zhao et al., 2018). In addition, ethyl heptanoate (OAV > 1) may also contribute to its fruity character.

Interestingly, the Mann–Whitney *U* test identified ten ester compounds exhibiting significant concentration differences between quality grades, seven of which were significantly enriched in premium-quality grade samples. Furthermore, the total concentration of quantified esters (*n* = 30) was significantly higher in premium samples (2.88 g/L, accounting for 75.75% of total volatile compounds) compared with normal samples (2.23 g/L, accounting for 71.97%). This trend aligns with previous findings (Chen et al., 2024). Therefore, the premium-quality grade samples were not only enriched with key aroma-active esters but also exhibited an overall higher ester content, which likely contributes to their more pronounced fruity and floral notes, ultimately enhancing overall sensory quality.

Alcohols are essential components of the flavor backbone in Baijiu, contributing to green, fruity, and mellow notes. While their individual aromas are typically less pronounced than esters, moderate concentrations of alcohols improve mouthfeel richness and flavor balance, thereby enhancing overall sensory perception (Wang et al., 2023). In this study, 12 alcohols were quantified, with the average total concentration being higher in premium-quality grade samples (448.13 mg/L) than in normal-quality grade samples (293.79 mg/L). Among these, 7 alcohols were significantly more abundant in premium samples, suggesting their potential role as markers distinguishing quality grades. Notably, 2-methylpropan-1-ol, *(E)*-hex-3-en-1-ol, butan-1-ol, and hexan-1-ol exhibited maximum OAVs exceeding 1, indicating measurable aroma activity and possible contributions to Baijiu flavor. In particular, butan-1-ol has been reported as a marker compound for differentiating *Nongxiangxing Baijiu* across regions (Song et al., 2020). Conversely, the lower alcohols content in normal-quality grade samples may result in a reduced body and thinner mouthfeel, which could negatively impact overall quality perception.

Several other highly aroma-active compounds (OAV > 100) also showed significantly higher concentrations in premium-quality grade samples. For example, *β*-damascenone, with an extremely low odor threshold and rose- and honey-like aroma (Niu et al., 2017), may enhance the floral complexity of premium Baijiu. Hexanal has been recognized as a key contributor to the fresh, green notes in Luzhou Laojiao (Chen et al., 2024), while 3-methylbutanal has been reported as a major contributor to pungent and spicy sensory attributes in Baijiu, potentially shaping its sharp and stimulating flavor profile (He et al., 2022).

Group B comprised aroma compounds that were present at significantly higher concentrations in normal-quality grade samples compared with premium-quality grade samples (*q* ≤ 0.05). A total of 11 compounds were identified, including 3 esters: ethyl 2-methylpropanoate, ethyl pentanoate, propyl hexanoate; 2 acids: acetic acid, butanoic acid; 2 alcohols: nonan-1-ol, octan-3-ol; 2 aldehydes: nonanal, *(2E,6Z)*-nona-2,6-dienal; and 2 aromatic compounds: 1-phenylethanone, phenylmethanol.

The Mann–Whitney *U* test identified three acids that exhibited significant concentration differences between quality grades, among which acetic acid and butanoic acid were significantly enriched in normal-quality grade samples. Both showed maximum OAVs >1, indicating that they are aroma-active compounds. The total concentration of acids (*n* = 10) in normal-quality samples reached 435.1 mg/L, significantly higher than that in premium-quality samples (292 mg/L). While moderate concentrations of acids can enhance flavor complexity and aftertaste, excessive acid levels may lead to undesirable sensory characteristics (Wei et al., 2020). Therefore, differences in acid concentrations may play an important role in the classification of *Nongxiangxing base Baijiu* quality grades.

One compound of particular interest was *(2E,6Z)*-nona-2,6-dienal, which was enriched in normal-quality samples. This compound is highly odor-active and has a characteristic cucumber-like note (Schieberle et al., 1990), which may contribute green and astringent off-notes when present at high concentrations.

**Fig. 5:**
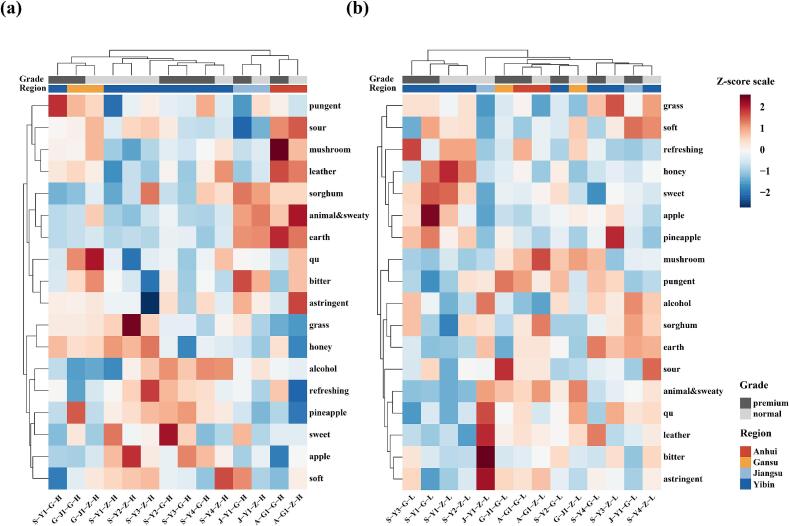
Clustering heatmap of different *Nongxiangxing base Baijiu* samples based on RATA sensory ratings. (a) 52% vol. (b) 42% vol.

To further explore whether differences in the concentrations of aroma-active compounds between quality grades contribute to sensory divergence, multiple statistical approaches were applied to screen for sensory attributes showing significant differences between grades. However, no attributes with strong discriminatory power were identified. Notably, sensory clustering analyses revealed a clear separation of samples based on production regions rather than quality grades (Fig. 5a), despite the significant chemical differences observed between grades.

### 3.4 Changes in the sensory profile of *Nongxiangxing base Baijiu* after ethanol reduction via water dilution

The alcohol content of *Nongxiangxing base Baijiu* typically exceeds 60% vol, which is overly potent for most consumers. To balance product style with consumer acceptability, Baijiu manufacturers commonly perform alcohol reduction during the blending stage, adjusting the final alcohol level to predetermined commercial targets (most commonly 52% vol, 42% vol, or lower). In practice, experienced blenders typically mix high-alcohol *Nongxiangxing base Baijiu* with lower-alcohol *Nongxiangxing base Baijiu* to achieve the desired alcohol content while minimizing alterations to the overall flavor profile. However, when a larger degree of reduction is required, deionized or soft water is added to further dilute ethanol levels.

To evaluate the impact of ethanol reduction by water on sensory characteristics, all samples were further diluted to 42% vol and evaluated using the Rate-All-That-Apply (RATA) method. Hierarchical clustering heatmaps were generated based on RATA sensory data for both the 52% vol and 42% vol samples (Fig. 5). At 52% vol, samples clustered clearly according to production region, while no distinct grouping was observed based on quality grades. After dilution to 42% vol, the regional clustering pattern became less pronounced. These results indicate that alcohol reduction alters, to some extent, the sensory expression of product style.

Furthermore, sensory radar plots comparing the 14 samples before and after dilution (Fig. A.1) revealed that although the concentrations of aroma compounds decreased proportionally from a chemical perspective, the perceived intensity of sensory attributes did not scale down linearly. This suggests a nonlinear relationship between compound concentration and sensory perception.

To statistically assess the effects of ethanol reduction on sensory properties, the Wilcoxon signed-rank test (two-tailed) was applied using paired data from the same samples before and after dilution (52% vol vs. 42% vol). The results (Table 4) showed that five sensory attributes exhibited significant differences after dilution, including a highly significant change in “alcohol” (*p* ≤ 0.01), and significant changes in “apple,” “astringent,” “grass,” and “sour” (*p* ≤ 0.05).

**Table 4:** Sensory attributes showing significant differences (*p* < 0.05) after dilution from 52% vol to 42% vol and the number of samples with decreased scores.

No.	Attribute	*p*-value^a^	Significance^b^	Number of Samples (*n* = 14)^c^
1	alcohol	0.001	***	2
2	apple	0.013	*	13
3	astringent	0.03	*	11
4	grass	0.035	*	11
5	sour	0.049	*	10

Note: ^a^ The *p*-values were determined using the Wilcoxon signed-rank test.

b Significance levels are indicated as follows: *p* < 0.05 (*), *p* < 0.01 (**), *p* < 0.001 (***).

c Number of samples showing decreased intensity for this sensory attribute after dilution.

Notably, after reduction from 52% vol to 42% vol, the intensities of the four sensory attributes “apple,” “grass,” “sour,” and “astringent” decreased in over 70% of the samples, whereas the perceived intensity of “alcohol” increased in more than 85% of the samples. Despite the overall decrease in aroma compound concentrations, the enhanced “alcohol” perception suggests that sensory intensity is not solely determined by compound concentration and may be influenced by additional factors.

## 4 General discussion

This study aimed to characterize regional and quality-grade differences in the chemosensory profiles of five-grain *Nongxiangxing base Baijiu* and to further evaluate the impact of ethanol reduction via water dilution.

Based on RATA sensory evaluation and quantitative analysis of volatile flavor compounds, we observed differences between Yibin and Non-Yibin samples at both the sensory-profile and chemical levels. In the region-discrimination PLS-DA model built using quantitative data of volatile compounds (*Q*^*2*^ = 0.685), 29 candidate discriminant compounds were identified (VIP > 1), indicating potential concentration differences between the Yibin and Non-Yibin groups. In contrast, sensory data provided limited discriminative power between the two groups. Although PERMANOVA indicated a significant between-group difference in the overall sensory profile (*p = 0.0007*), Mann–Whitney *U* tests did not identify any individual sensory attributes with statistically significant differences. We therefore supplemented effect size (Cliff's δ) and screened 10 potentially differential sensory attributes using |Cliff's δ| ≥ 0.47 (large effect) as the threshold, suggesting that these attributes may potentially differ between Yibin and Non-Yibin samples.

Linking chemical composition to sensory attributes, the enrichment of esters in Yibin samples may relate to their higher scores on fruit-associated attributes such as “apple” and “pineapple” (He et al., 2020). In addition, Yibin samples contained higher levels of (methyltrisulfanyl)methane (DMTS). Although DMTS itself does not exhibit a fruity character, it has been reported to indirectly enhance fruitiness through interactions with other aroma components (Lytra et al., 2012). The higher levels of alcohols in Yibin samples may also contribute to their higher ratings on the “alcohol” attribute. By contrast, the Non-Yibin samples showed higher levels of octan-2-one and *(E)*-oct-3-en-2-one, both of which have earthy and mushroom odor characteristics (Sun et al., 2024) and may be associated with the higher scores for “earth” and “mushroom”.

Notably, the attributes that scored higher in Yibin samples (“apple,” “refreshing,” “pineapple,” and “alcohol”) showed highly significant positive correlations in our previous work with expert-assessor descriptors (“jiao,” “mellow,” and “grain”), whereas the attributes that scored lower (“animal & sweaty,” “earth,” “bitter,” “qu,” “mushroom,” and “leather”) showed highly significant positive correlations with the expert descriptor “mud” (Xiangli et al., 2024). Using expert-assessor terminology as an auxiliary interpretive framework, these correlations suggest that Yibin samples exhibit stronger “jiao,” “mellow,” and “grain”-related features but comparatively weaker “mud”-related characteristics than samples from other regions. This inference is broadly consistent with previous studies on regional flavor differentiation of *Nongxiangxing Baijiu* (Dong et al., 2018; He et al., 2020; Song et al., 2020; Wang et al., 2025). “jiao” is a typical aroma characteristic of *Nongxiangxing Baijiu*, and ethyl hexanoate has been demonstrated to be one of the key odorants contributing to “jiao” (Dai et al., 2026). In this study, the Yibin samples contained higher levels of ethyl hexanoate than those from other regions; therefore, we speculate that this compound may be one of the main reasons for the stronger “jiao”-related character in the Yibin samples. The formation of ethyl hexanoate is closely related to the level of its precursor, hexanoic acid. Previous research has shown that aged pits in the Yibin region are enriched in hexanoic-acid-producing functional microbial communities, which increases hexanoic acid levels and thereby promotes the formation and accumulation of ethyl hexanoate (Hu et al., 2015). In addition, “animal,” “earth,” “mushroom,” and “leather”-like notes are often considered to be associated with pit mud. Within the scope of the samples included in this study, pits in the Non-Yibin regions were smaller than those in the Yibin region, resulting in a relatively larger contact area between Jiupei (fermented grains) and pit mud during fermentation, which may facilitate the enrichment of pit-mud-associated flavor compounds. This may be one of the reasons why the Non-Yibin samples showed higher scores for these pit-mud-related sensory attributes. Nevertheless, because none of the individual sensory attributes showed statistically significant between-group differences in this study, these explanations remain speculative and should be validated using larger sample sets.

By comparing premium-quality grade and normal-quality grade samples from different manufacturers across production regions, we identified significant grade-related differences in the concentrations of multiple volatile components, and these differences were largely consistent across regions. Although the current sample size is still insufficient to propose universal, cross-regional chemical criteria for quality grading, the shared chemical features identified in this study provide preliminary evidence toward elucidating the material basis underlying base Baijiu quality.

Notably, although the two quality grades differed significantly at the chemical level, these differences did not consistently translate into clear sensory separation. Because no mechanistic experiments were conducted, we propose the following plausible explanations based on the available evidence. (1) The overall aroma profile may depend more on the relative ratios among a limited set of key odorants than on absolute concentrations (Ma et al., 2025). Thus, within the same production region, sensory profiles may converge when the proportional relationships among key odorants remain relatively stable, even if the concentrations of certain components fluctuate significantly. (2) Matrix components may form an “aroma-buffering system,” such that concentration changes in some odorants are insufficient to overcome this buffering effect (Ferreira et al., 2022). Moreover, human odor-intensity perception often follows an S-shaped concentration-intensity relationship (Baldovini & Chaintreau, 2020), and if concentration changes mainly occur within low-sensitivity ranges, even significant concentration differences may be difficult to perceive consistently. (3) Quality grading likely emphasizes holistic characteristics (e.g., body fullness, harmony, and aroma complexity), which require integrative judgment and extensive professional experience. Although our panelists underwent strict selection and training, reliably distinguishing subtle variations in these multidimensional attributes remains challenging. (4) Although the RATA lexicon covered the sensory characteristics of the samples as comprehensively as possible, some complex flavor experiences associated with quality grade are difficult to externalize into consensus descriptors shared across panelists, making such differences hard to capture in the evaluation system. Based on these results, we emphasize that if quality grades are difficult to distinguish at the sensory level, or if differences cannot be clearly articulated in sensory language, establishing objective and quantifiable, chemistry-based criteria becomes even more necessary; this constitutes one of the key significances of this study.

Finally, we reduced the ethanol content of the samples from 52% vol to 42% vol by water dilution to evaluate the impact of dilution on sensory profiles. The results showed that, although compound concentrations decreased proportionally during dilution, the intensities of sensory attributes did not decrease accordingly. Notably, the “alcohol” attribute increased in more than 85% of the samples. This indicates that changes in flavor profiles do not follow a simple linear response to changes in overall compound concentrations. Therefore, our earlier proposition that the overall aroma profile may be driven more by the relative proportions among a limited set of key odorants than by absolute concentrations does not necessarily hold when the matrix is altered. We speculate that one reason is that changes in matrix ethanol concentration can shift the relative release of volatile compounds, thereby altering aroma perception (Le Quéré & Schoumacker, 2023). A more critical reason is that matrix ethanol concentration can substantially affect olfactory thresholds. Previous work has shown that, as ethanol concentration decreases, the odor thresholds of most volatile compounds decrease, and the magnitude of decrease varies across compounds (Zhang, Lu, Yu, Chen and Xu, 2025a). Accordingly, we hypothesize that during dilution, the odor thresholds of certain compounds decrease more markedly, leading to a faster increase in their OAVs. As a result, these compounds become more perceptually prominent, ultimately increasing the prominence of their associated attributes, such as “alcohol” in the sensory profile.

Although this study provides valuable preliminary evidence for flavor differences in *Nongxiangxing base Baijiu* across production regions and quality grades, as noted above, the conclusions cannot be further strengthened due to limitations in sample coverage and the precision of sensory evaluation. Future work will focus on four directions: (1) expanding the sample size to develop more robust and generalizable discrimination models; (2) increasing the number of panelists and strengthening training to improve the stability and reproducibility of sensory data; (3) conducting mechanistic validation of the key hypotheses proposed in this study to move from phenomenological observations to mechanistic understanding; (4) introducing machine-learning approaches to capture non-linear relationships between sensory attributes and compound concentrations, thereby improving the modeling capacity to predict sensory performance from chemical data.

## 5 Conclusions

This study systematically characterized the sensory profiles of *Nongxiangxing base Baijiu* across production regions and quality grades, and quantified a total of 96 aroma-active compounds. Differences were observed between samples from the Yibin region and those from other regions in both overall sensory profiles and chemical composition: based on effect-size screening, Yibin samples tended to show higher scores for “apple,” “refreshing,” “pineapple,” and “alcohol,” but lower scores for “animal & sweaty,” “earth,” “bitter,” “qu,” “mushroom,” and “leather.” A PLS-DA model built on quantitative volatile-compound data identified 29 candidate discriminant compounds with VIP > 1, suggesting potential concentration differences between the Yibin and Non-Yibin samples; among them, alcohols, esters, and pyrazines tended to be higher in the Yibin samples.

Hierarchical clustering suggested compositional separation between premium-quality grade and normal-quality grade samples, with similar patterns observed across the sampled regions and manufacturers. Furthermore, Mann–Whitney *U* tests identified 34 compounds with significant concentration differences between quality grades: esters and alcohols were significantly enriched in premium-quality grade samples, whereas acids were more abundant in normal-quality grade samples.

To evaluate the sensory impact of ethanol reduction via water dilution, all samples were diluted from 52% vol to 42% vol, and sensory profiles before and after dilution were compared. The results showed that ethanol reduction altered sensory expression to some extent, and changes in sensory profiles did not correspond linearly to the proportional dilution of aroma-compound concentrations.

Overall, this study provides valuable data to further refine the concept of regional differentiation in *Nongxiangxing base Baijiu* and to support a shift in quality grading from relatively subjective, experience-based judgments toward an objective, quantifiable evaluation framework. In addition, our findings highlight the need to develop ethanol-reduction strategies that maintain style stability, thereby improving drinkability while preserving overall flavor quality.

## CRediT authorship contribution statement

**Ruotao Zhou:** Writing – original draft, Visualization, Methodology, Investigation. **Ran Xiangli:** Methodology, Investigation, Formal analysis, Conceptualization. **Dong Zhao:** Supervision, Resources, Conceptualization. **Jia Zheng:** Resources, Methodology, Conceptualization. **Jian Su:** Resources, Methodology, Conceptualization. **Zheng Feng:** Resources, Methodology, Conceptualization. **Yue Ma:** Supervision, Methodology, Funding acquisition, Formal analysis, Conceptualization. **Yan Xu:** Supervision, Funding acquisition, Conceptualization.

## Funding sources

This work was supported by the 10.13039/501100001809National Natural Science Foundation of China for Young Scholars [32202218]; the 10.13039/501100012226Fundamental Research Funds for the Central Universities [JUSRP123038]; the Postgraduate Research & Practice Innovation Program of Jiangsu Province [SJCX25_1341].

## Declaration of competing interest

The authors declare that they have no known competing financial interests or personal relationships that could have appeared to influence the work reported in this paper.

Acknowledgments

The authors gratefully acknowledge all the participants who took part in the sensory experiments.

## Data Availability

Data will be made available on request.

## Appendix A Supplementary data

Supplementary material 1 Fig. A.1. Sensory radar charts of different Baijiu samples at 42% vol and 52% vol. Supplementary material 1

Supplementary material 2 Table A.1. Information on chemical internal standards; Table A.2. Modified intensity of sensory attributes of *Nongxiangxing base Baijiu* evaluated by RATA analysis; and Table A.3. Quantification results of odor-active compounds in different Baijiu samples. Supplementary material 2
