# Supplementary material for: Chemical signatures and sensory perception of Nongxiangxing Baijiu: regional and quality-grade discrimination and the modulatory role of ethanol
Source: Food Chem X. 2026 Mar 26;35:103790. doi: 10.1016/j.fochx.2026.103790 (PMC13081665; doi:10.1016/j.fochx.2026.103790)
Supplement: Supplementary material 1 — Fig. A.1. Sensory radar charts of different Baijiu samples at 42% vol and 52% vol. [file mmc1.docx]

Fig. A.1 Radar chart comparison of sensory attributes before and after alcohol dilution across Baijiu samples


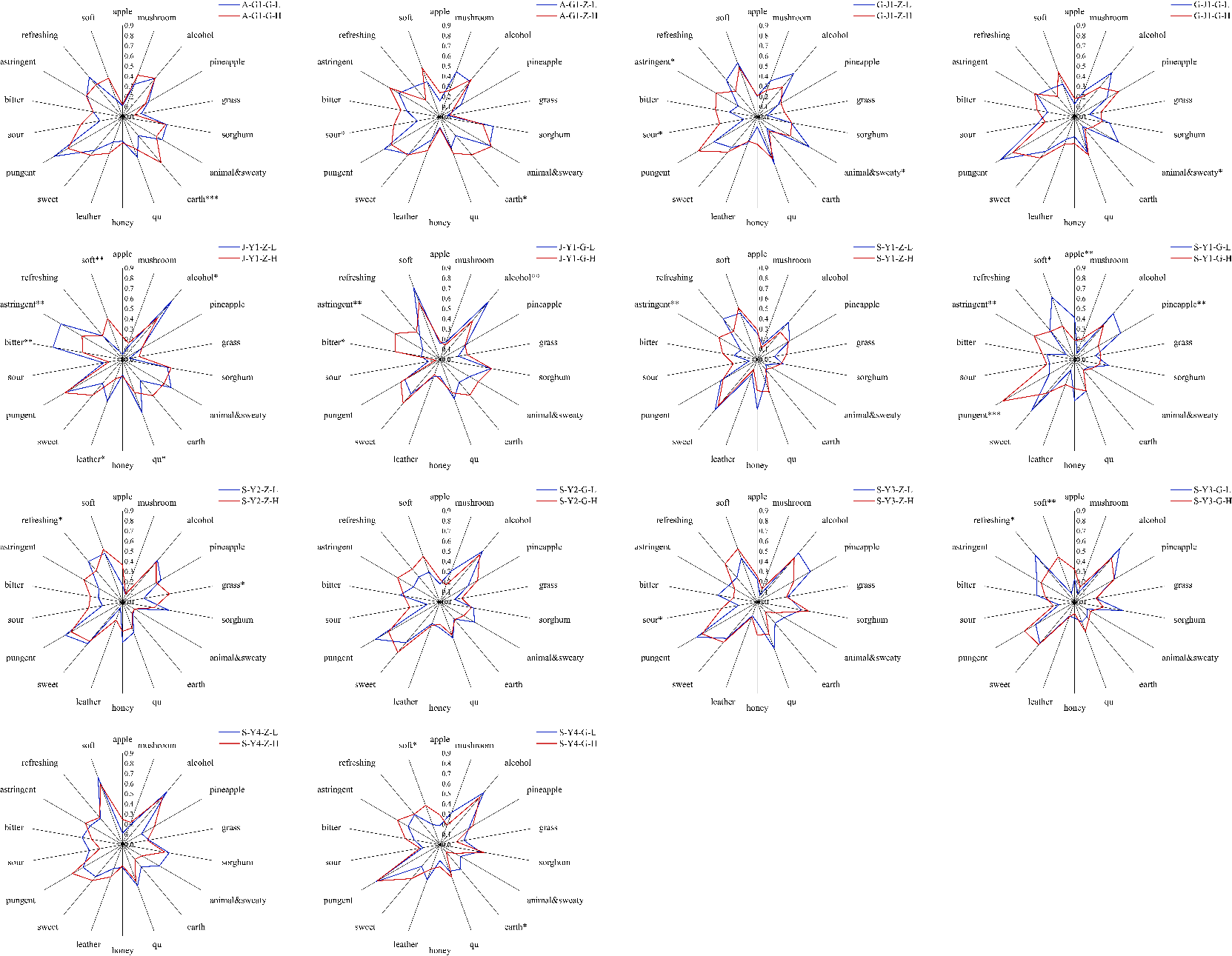


Note: the significant values were determined using the Wilcoxon Signed-Rank Test.
