# Supplementary material for: Chemical signatures and sensory perception of Nongxiangxing Baijiu: regional and quality-grade discrimination and the modulatory role of ethanol
Source: Food Chem X. 2026 Mar 26;35:103790. doi: 10.1016/j.fochx.2026.103790 (PMC13081665; doi:10.1016/j.fochx.2026.103790)
Supplement: Supplementary material 2 — Table A.1. Information on chemical internal standards; Table A.2. Modified intensity of sensory attributes of Nongxiangxing base Baijiu evaluated by RATA analysis; and Table A.3. Quantification results of odor-active compounds in different Baijiu samples. [file mmc2.docx]

**Supplementary Table 1 Information of chemical internal standards**

| No. | compound | concentration（mg/L） | purity | company |
| --- | --- | --- | --- | --- |
| IS1 | 2-methylbutan-2-ol | 2.33 | chromatographic grade (purity ≥97%) | Sigma Aldrich Trading Co., Ltd. (Shanghai, China) |
| IS2 | pentyl acetate | 2.64 | chromatographic grade (purity ≥97%) | Sigma Aldrich Trading Co., Ltd. (Shanghai, China) |
| IS3 | 3,3-dimethylbutanoic acid | 200.02 | chromatographic grade (purity ≥97%) | Sigma Aldrich Trading Co., Ltd. (Shanghai, China) |
| IS4 | guaiacol-*d_3_* | 49.99 | chromatographic grade (purity ≥97%) | Sigma Aldrich Trading Co., Ltd. (Shanghai, China) |
| IS5 | furfural-*d_4_* | 237.5 | chromatographic grade (purity ≥97%) | Sigma Aldrich Trading Co., Ltd. (Shanghai, China) |
| IS6 | 2-methoxy-3-(2-methylpropyl)pyrazine | 200.06 | chromatographic grade (purity ≥97%) | Sigma Aldrich Trading Co., Ltd. (Shanghai, China) |
| IS7 | hexan-1-ol-*d_13_* | 162.7 | chromatographic grade (purity ≥97%) | Sigma Aldrich Trading Co., Ltd. (Shanghai, China) |
| IS8 | ethyl octanoate-*d_15_* | 988.05 | chromatographic grade (purity ≥97%) | Sigma Aldrich Trading Co., Ltd. (Shanghai, China) |
| IS9 | octanal-*d_16_* | 500.02 | chromatographic grade (purity ≥97%) | Sigma Aldrich Trading Co., Ltd. (Shanghai, China) |
| IS10 | phenylethyl acetate-*d_3_* | 247.01 | chromatographic grade (purity ≥97%) | Sigma Aldrich Trading Co., Ltd. (Shanghai, China) |
| IS11 | acetophenone-*d_3_* | 247.5 | chromatographic grade (purity ≥97%) | Sigma Aldrich Trading Co., Ltd. (Shanghai, China) |
| IS12 | isopropyl disulfide | 12.96 | chromatographic grade (purity ≥97%) | Sigma Aldrich Trading Co., Ltd. (Shanghai, China) |

**Supplementary Table 2 Sensory attribute intensity of *Nongxiangxing* base baijiu (modified RATA calculation method)**

| attributes | modified intensity value ^a^ | | | | | | | | | | | | | |
| --- | --- | --- | --- | --- | --- | --- | --- | --- | --- | --- | --- | --- | --- | --- |
|  | A-G1-G-L | A-G1-Z-L | G-J1-Z-L | G-J1-G-L | J-Y1-Z-L | J-Y1-G-L | S-Y1-Z-L | S-Y1-G-L | S-Y2-Z-L | S-Y2-G-L | S-Y3-Z-L | S-Y3-G-L | S-Y4-Z-L | S-Y4-G-L |
| apple | 0.11 | 0.16 | 0.20 | 0.13 | 0.05 | 0.16 | 0.26 | 0.41 | 0.16 | 0.18 | 0.23 | 0.23 | 0.11 | 0.19 |
| mushroom^***^ | 0.34 | 0.47 | 0.38 | 0.27 | 0.12 | 0.18 | 0.16 | 0.07 | 0.08 | 0.33 | 0.07 | 0.09 | 0.20 | 0.34 |
| alcohol^***^ | 0.49 | 0.46 | 0.56 | 0.57 | 0.76 | 0.74 | 0.48 | 0.59 | 0.53 | 0.65 | 0.63 | 0.69 | 0.68 | 0.67 |
| pineapple^**^ | 0.29 | 0.24 | 0.24 | 0.37 | 0.21 | 0.30 | 0.37 | 0.52 | 0.43 | 0.32 | 0.60 | 0.45 | 0.22 | 0.33 |
| grass | 0.19 | 0.07 | 0.10 | 0.15 | 0.07 | 0.18 | 0.17 | 0.21 | 0.21 | 0.15 | 0.31 | 0.21 | 0.26 | 0.24 |
| sorghum | 0.44 | 0.53 | 0.34 | 0.35 | 0.45 | 0.51 | 0.26 | 0.34 | 0.46 | 0.34 | 0.44 | 0.48 | 0.46 | 0.40 |
| animal&sweaty^***^ | 0.45 | 0.57 | 0.59 | 0.50 | 0.55 | 0.34 | 0.10 | 0.15 | 0.13 | 0.39 | 0.31 | 0.14 | 0.42 | 0.23 |
| earth^***^ | 0.24 | 0.24 | 0.15 | 0.12 | 0.27 | 0.29 | 0.14 | 0.13 | 0.16 | 0.22 | 0.27 | 0.19 | 0.29 | 0.32 |
| qu | 0.42 | 0.32 | 0.49 | 0.37 | 0.56 | 0.41 | 0.23 | 0.33 | 0.32 | 0.38 | 0.48 | 0.21 | 0.43 | 0.28 |
| honey^*^ | 0.24 | 0.10 | 0.10 | 0.20 | 0.15 | 0.16 | 0.49 | 0.41 | 0.40 | 0.26 | 0.20 | 0.16 | 0.23 | 0.16 |
| leather^**^ | 0.26 | 0.24 | 0.28 | 0.23 | 0.44 | 0.18 | 0.15 | 0.11 | 0.06 | 0.23 | 0.14 | 0.15 | 0.25 | 0.37 |
| sweet | 0.43 | 0.48 | 0.39 | 0.44 | 0.31 | 0.44 | 0.65 | 0.66 | 0.53 | 0.52 | 0.47 | 0.53 | 0.41 | 0.28 |
| pungent^***^ | 0.78 | 0.63 | 0.49 | 0.84 | 0.63 | 0.40 | 0.41 | 0.29 | 0.65 | 0.73 | 0.69 | 0.44 | 0.45 | 0.72 |
| sour^***^ | 0.23 | 0.23 | 0.13 | 0.37 | 0.19 | 0.12 | 0.08 | 0.25 | 0.20 | 0.13 | 0.19 | 0.16 | 0.33 | 0.17 |
| bitter^***^ | 0.29 | 0.33 | 0.28 | 0.27 | 0.69 | 0.19 | 0.23 | 0.28 | 0.24 | 0.40 | 0.39 | 0.38 | 0.40 | 0.26 |
| astringent^***^ | 0.41 | 0.47 | 0.22 | 0.43 | 0.70 | 0.20 | 0.19 | 0.10 | 0.27 | 0.30 | 0.33 | 0.43 | 0.37 | 0.36 |
| refreshing | 0.51 | 0.39 | 0.47 | 0.36 | 0.30 | 0.31 | 0.52 | 0.38 | 0.52 | 0.32 | 0.31 | 0.60 | 0.34 | 0.39 |
| soft^***^ | 0.18 | 0.37 | 0.57 | 0.34 | 0.09 | 0.75 | 0.49 | 0.65 | 0.51 | 0.31 | 0.46 | 0.09 | 0.71 | 0.21 |

Note: ^a^RATA data in this study were processed according to the method of (Xiangli et al., 2024), instead of the traditional mean value calculation.

^b^The Kruskal-Wallis test was applied to the raw RATA data to identify sensory attributes exhibiting significant differences among the various baijiu samples. significant (*p* < 0.05, *), very significant (*p* < 0.01, **), and extremely significant (*p* < 0.001, ***).

**Supplementary Table 2 (continued) Sensory attribute intensity of *Nongxiangxing* base baijiu (modified RATA calculation method)**

| attributes | modified intensity value ^a^ | | | | | | | | | | | | | | *p* value ^b^ |
| --- | --- | --- | --- | --- | --- | --- | --- | --- | --- | --- | --- | --- | --- | --- | --- |
|  | A-G1-G-H | A-G1-Z-H | G-J1-Z-H | G-J1-G-H | J-Y1-Z-H | J-Y1-G-H | S-Y1-Z-H | S-Y1-G-H | S-Y2-Z-H | S-Y2-G-H | S-Y3-Z-H | S-Y3-G-H | S-Y4-Z-H | S-Y4-G-H |  |
| apple | 0.12 | 0.24 | 0.21 | 0.18 | 0.23 | 0.18 | 0.29 | 0.19 | 0.37 | 0.23 | 0.26 | 0.32 | 0.25 | 0.29 | 0.371 |
| mushroom^***^ | 0.44 | 0.28 | 0.28 | 0.21 | 0.17 | 0.15 | 0.12 | 0.22 | 0.08 | 0.18 | 0.14 | 0.16 | 0.23 | 0.20 | 0.000 |
| alcohol^***^ | 0.50 | 0.48 | 0.38 | 0.38 | 0.53 | 0.50 | 0.35 | 0.44 | 0.52 | 0.61 | 0.57 | 0.57 | 0.61 | 0.61 | 0.000 |
| pineapple^**^ | 0.26 | 0.15 | 0.27 | 0.49 | 0.22 | 0.29 | 0.35 | 0.27 | 0.38 | 0.43 | 0.42 | 0.45 | 0.36 | 0.37 | 0.003 |
| grass | 0.12 | 0.10 | 0.27 | 0.26 | 0.16 | 0.27 | 0.29 | 0.26 | 0.47 | 0.22 | 0.29 | 0.22 | 0.25 | 0.16 | 0.072 |
| sorghum | 0.43 | 0.43 | 0.35 | 0.27 | 0.48 | 0.52 | 0.24 | 0.25 | 0.32 | 0.32 | 0.52 | 0.29 | 0.42 | 0.44 | 0.093 |
| animal&sweaty^***^ | 0.38 | 0.58 | 0.38 | 0.20 | 0.47 | 0.44 | 0.16 | 0.19 | 0.13 | 0.27 | 0.21 | 0.18 | 0.22 | 0.17 | 0.000 |
| earth^***^ | 0.59 | 0.49 | 0.18 | 0.16 | 0.46 | 0.46 | 0.13 | 0.13 | 0.15 | 0.23 | 0.13 | 0.22 | 0.19 | 0.10 | 0.000 |
| qu | 0.33 | 0.38 | 0.44 | 0.40 | 0.35 | 0.35 | 0.34 | 0.33 | 0.27 | 0.34 | 0.34 | 0.31 | 0.38 | 0.34 | 0.163 |
| honey^*^ | 0.26 | 0.11 | 0.27 | 0.26 | 0.16 | 0.20 | 0.31 | 0.29 | 0.29 | 0.22 | 0.32 | 0.11 | 0.21 | 0.22 | 0.013 |
| leather^**^ | 0.38 | 0.35 | 0.26 | 0.29 | 0.20 | 0.16 | 0.10 | 0.26 | 0.19 | 0.25 | 0.16 | 0.16 | 0.34 | 0.26 | 0.005 |
| sweet | 0.49 | 0.49 | 0.45 | 0.53 | 0.46 | 0.56 | 0.60 | 0.44 | 0.50 | 0.65 | 0.52 | 0.55 | 0.46 | 0.44 | 0.194 |
| pungent^***^ | 0.62 | 0.55 | 0.67 | 0.70 | 0.66 | 0.44 | 0.39 | 0.82 | 0.59 | 0.58 | 0.63 | 0.57 | 0.57 | 0.71 | 0.000 |
| sour^***^ | 0.41 | 0.46 | 0.38 | 0.29 | 0.14 | 0.04 | 0.24 | 0.28 | 0.34 | 0.30 | 0.35 | 0.21 | 0.23 | 0.20 | 0.000 |
| bitter^***^ | 0.29 | 0.39 | 0.42 | 0.37 | 0.40 | 0.44 | 0.35 | 0.32 | 0.32 | 0.36 | 0.24 | 0.29 | 0.31 | 0.35 | 0.001 |
| astringent^***^ | 0.41 | 0.57 | 0.46 | 0.45 | 0.46 | 0.51 | 0.43 | 0.46 | 0.44 | 0.48 | 0.26 | 0.38 | 0.42 | 0.48 | 0.000 |
| refreshing | 0.41 | 0.22 | 0.33 | 0.26 | 0.31 | 0.36 | 0.36 | 0.36 | 0.40 | 0.42 | 0.49 | 0.40 | 0.35 | 0.39 | 0.376 |
| soft^***^ | 0.41 | 0.51 | 0.52 | 0.47 | 0.43 | 0.59 | 0.54 | 0.35 | 0.55 | 0.48 | 0.56 | 0.47 | 0.63 | 0.41 | 0.00 |

**Supplementary Table 3 Quantitative data of odor-active compounds (52% vol)**

| No. | CAS | Compounds | Threshold (μg/L) | Concentration (μg/L) | | | | | |
| --- | --- | --- | --- | --- | --- | --- | --- | --- | --- |
|  |  |  |  | A-G1-G-Mean | A-G1-G-SD | G-J1-G-Mean | G-J1-G-SD | J-Y1-G-Mean | J-Y1-G-SD |
| 1 | 105-57-7 | 1,1-diethoxyethane | 2090 ^a^ | 129300.6 | 43.9 | 110853.9 | 61.2 | 154678.5 | 363.4 |
| 2 | 141-78-6 | ethyl acetate | 32552 ^a^ | 1154413.8 | 205.1 | 972104.4 | 328.0 | 1212255.7 | 171.5 |
| 3 | 97-62-1 | ethyl 2-methylpropanoate | 57.5 ^a^ | 3742.6 | 11.6 | 4365.8 | 24.3 | 3962.5 | 12.9 |
| 4 | 110-62-3 | pentanal | 725 ^d^ | 5091.5 | 22.0 | 3233.9 | 15.4 | 4184.4 | 11.7 |
| 5 | 78-92-2 | butan-2-ol | 50000 ^m^ | 149502.7 | 200.6 | 144618.9 | 335.9 | 143609.2 | 297.2 |
| 6 | 110-19-0 | 2-methylpropyl acetate | 922 ^a^ | 4262.0 | 8.2 | 2077.4 | 5.2 | 2471.0 | 23.3 |
| 7 | 105-54-4 | ethyl butanoate | 81.5 ^a^ | 92827.1 | 98.3 | 98326.3 | 355.2 | 150732.4 | 111.5 |
| 8 | 7452-79-1 | ethyl 2-methylbutanoate | 18.0 ^a^ | 1775.3 | 12.0 | 1556.2 | 10.0 | 1772.1 | 8.0 |
| 9 | 108-64-5 | ethyl 3-methylbutanoate | 6.89 ^a^ | 6526.2 | 21.5 | 5336.6 | 4.5 | 8237.9 | 96.5 |
| 10 | 590-86-3 | 3-methylbutanal | 16.5 ^a^ | 8236.5 | 20.1 | 13265.2 | 26.8 | 10813.3 | 16.3 |
| 11 | 123-86-4 | butyl acetate | 1800 ^h^ | 3720.3 | 18.9 | 3148.0 | 38.5 | 2480.3 | 8.5 |
| 12 | 66-25-1 | hexanal | 25.5 ^a^ | 13825.9 | 186.8 | 12504.5 | 27.8 | 10829.5 | 22.5 |
| 13 | 78-83-1 | 2-methylpropan-1-ol | 28300 ^f^ | 77880.9 | 23.6 | 49554.2 | 23.4 | 87342.5 | 48.6 |
| 14 | 71-36-3 | butan-1-ol | 2733 ^a^ | 19366.1 | 28.2 | 12216.3 | 5.8 | 17531.4 | 15.6 |
| 15 | 6032-29-7 | pentan-2-ol | 194313 ^a^ | 43480.6 | 31.0 | 25735.4 | 59.1 | 48406.4 | 49.6 |
| 16 | 123-92-2 | 3-methylbutyl acetate | 93.9 ^a^ | 1269.8 | 19.2 | 1219.7 | 9.7 | 238.2 | 3.4 |
| 17 | 539-82-2 | ethyl pentanoate | 26.8 ^a^ | 10865.2 | 200.4 | 9033.9 | 38.7 | 8270.7 | 13.9 |
| 18 | 123-51-3 | 3-methylbutan-1-ol | 179191 ^a^ | 129621.8 | 273.6 | 177419.5 | 345.7 | 106182.0 | 1676.1 |
| 19 | 123-66-0 | ethyl hexanoate | 55.3 ^a^ | 1453523.7 | 34596.2 | 1349136.3 | 1397.9 | 1862892.7 | 73.8 |
| 20 | 109-08-0 | 2-methylpyrazine | 60000 ^a^ | 656.8 | 3.1 | 826.6 | 5.5 | 927.9 | 8.7 |
| 21 | 142-92-7 | hexyl acetate | 5560 ^d^ | 513.7 | 9.9 | 1114.8 | 9.5 | 513.4 | 14.0 |
| 22 | 106-27-4 | 3-methylbutyl butanoate | 915 ^f^ | 1224.6 | 14.6 | 1003.6 | 8.6 | 878.4 | 1.9 |
| 23 | 108-50-9 | 2,6-dimethylpyrazine | 791 ^a^ | 94.3 | 0.7 | 117.8 | 1.0 | 132.6 | 1.2 |
| 24 | 111-13-7 | octan-2-one | 250 ^l^ | 17.5 | 4.2 | 23.7 | 0.5 | 7.0 | 0.0 |
| 25 | 659-70-1 | 3-methylbutyl 3-methylbutanoate | 134 ^b^ | 28.5 | 0.2 | 18.2 | 0.1 | 27.4 | 0.3 |
| 26 | 626-77-7 | propyl hexanoate | 12784 ^a^ | 3291.5 | 112.2 | 3222.7 | 4.6 | 2232.9 | 2.5 |
| 27 | 124-13-0 | octanal | 39.6 ^a^ | 5539.7 | 42.4 | 5324.4 | 6.0 | 7263.7 | 60.2 |
| 28 | 543-49-7 | heptan-2-ol | 1431 ^a^ | 23404.1 | 355.9 | 6300.8 | 22.9 | 12243.5 | 173.0 |
| 29 | 106-30-9 | ethyl heptanoate | 13153 ^a^ | 44221.9 | 66.8 | 25041.8 | 22.9 | 31290.9 | 18.0 |
| 30 | 105-79-3 | 2-methylpropyl hexanoate | 5350 ^d^ | 41.4 | 0.4 | 85.4 | 2.3 | 168.7 | 9.3 |
| 31 | 111-27-3 | hexan-1-ol | 5370 ^d^ | 20051.7 | 7.3 | 21966.6 | 13.4 | 19465.1 | 7.5 |
| 32 | 928-97-2 | (E)-hex-3-en-1-ol | 400 ^n^ | 167.2 | 4.9 | 199.9 | 2.1 | 338.6 | 2.9 |
| 33 | 821-55-6 | nonan-2-one | 393 ^d^ | 127.0 | 6.1 | 500.9 | 8.7 | 1769.3 | 6.6 |
| 34 | 589-98-0 | octan-3-ol | 483 ^a^ | 160.8 | 4.8 | 74.9 | 4.4 | 129.7 | 1.1 |
| 35 | 13925-03-6 | 2-ethyl-6-methylpyrazine | 40.0 ^d^ | 5.6 | 0.1 | 7.4 | 0.2 | 29.9 | 0.4 |
| 36 | 3658-80-8 | (methyltrisulfanyl)methane | 0.36 ^a^ | 87.5 | 0.5 | 86.3 | 1.7 | 46.5 | 1.0 |
| 37 | 1669-44-9 | (E)-oct-3-en-2-one | 48.2 ^b^ | 676.9 | 24.9 | 388.0 | 10.0 | 55.8 | 0.9 |
| 38 | 13067-27-1 | 2,6-diethylpyrazine | 6.00 ^d^ | 2.3 | 0.1 | 0.6 | 0.1 | 1.0 | 0.0 |
| 39 | 14667-55-1 | 2,3,5-trimethylpyrazine | 730 ^a^ | 12.4 | 0.3 | 6.5 | 0.0 | 8.7 | 0.0 |
| 40 | 124-19-6 | nonanal | 122 ^a^ | 177.3 | 1.7 | 131.7 | 2.0 | 570.0 | 7.0 |
| 41 | 64-19-7 | acetic acid | 160000 ^f^ | 119295.5 | 149.2 | 55799.0 | 14.8 | 44887.6 | 3.2 |
| 42 | 106-32-1 | ethyl octanoate | 12.9 ^a^ | 12988.7 | 13.2 | 9754.9 | 25.2 | 15934.3 | 6.6 |
| 43 | 111-70-6 | heptan-1-ol | 26600 ^i^ | 1707.7 | 37.3 | 5458.5 | 7.3 | 2796.3 | 21.2 |
| 44 | 7779-80-8 | 2-methylpropyl heptanoate | 26264 ^d^ | 9.3 | 0.1 | 18.1 | 0.4 | 8.0 | 0.1 |
| 45 | 2198-61-0 | 3-methylbutyl hexanoate | 1400 ^e^ | 627.1 | 4.3 | 778.6 | 7.7 | 1502.8 | 11.0 |
| 46 | 13529-27-6 | 2-(diethoxymethyl)furan | 6172 ^f^ | 8329.7 | 54.9 | 4180.1 | 5.1 | 3726.6 | 18.6 |
| 47 | 585-24-0 | 2-methylpropyl 2-hydroxypropanoate | 4904 ^b^ | 1884.4 | 13.1 | 2061.0 | 38.8 | 2112.3 | 11.2 |
| 48 | 1124-11-4 | 2,3,5,6-tetramethylpyrazine | 80073 ^a^ | 90.0 | 2.6 | 71.9 | 0.8 | 247.0 | 4.2 |
| 49 | 98-01-1 | furan-2-carbaldehyde | 44000 ^a^ | 1978.7 | 3.6 | 1745.1 | 2.1 | 2638.8 | 368.4 |
| 50 | 540-07-8 | pentyl hexanoate | 13802 ^d^ | 1096.4 | 8.9 | 2004.5 | 76.0 | 618.8 | 1.9 |
| 51 | 112-31-2 | decanal | 70.8 ^e^ | 1436.4 | 19.9 | 392.8 | 2.6 | 401.5 | 2.1 |
| 52 | 100-52-7 | benzaldehyde | 4203 ^a^ | 4484.8 | 8.6 | 2127.1 | 48.7 | 2498.4 | 9.1 |
| 53 | 624-13-5 | propyl octanoate | 662 ^a^ | 193.3 | 2.3 | 212.8 | 4.2 | 31.3 | 1.3 |
| 54 | 123-29-5 | ethyl nonanoate | 3151 ^a^ | 5934.8 | 29.9 | 4117.0 | 97.9 | 10615.0 | 49.6 |
| 55 | 79-09-4 | propanoic acid | 18200 ^a^ | 3408.1 | 6.1 | 13348.6 | 6.2 | 8186.8 | 24.7 |
| 56 | 623-17-6 | furan-2-ylmethyl acetate | 26050 ^p^ | 52.7 | 0.3 | 37.2 | 0.5 | 94.8 | 0.6 |
| 57 | 108-29-2 | 5-methyloxolan-2-one | 2200 ^a^ | 6.6 | 0.5 | 7.8 | 0.0 | 0.0 | 0.0 |
| 58 | 111-87-5 | octan-1-ol | 1100 ^d^ | 134.3 | 1.6 | 145.1 | 1.2 | 229.5 | 1.2 |
| 59 | 79-31-2 | 2-methylpropanoic acid | 1580 ^f^ | 46374.0 | 39.7 | 16339.3 | 32.5 | 6332.4 | 3.9 |
| 60 | 557-48-2 | (2E,6Z)-nona-2,6-dienal | 0.64 ^b^ | 145.5 | 1.2 | 180.4 | 2.1 | 881.8 | 7.1 |
| 61 | 6378-65-0 | hexyl hexanoate | 1891 ^d^ | 2390.9 | 12.9 | 2928.4 | 8.8 | 2520.8 | 7.3 |
| 62 | 107-92-6 | butanoic acid | 965 ^a^ | 11654.9 | 22.4 | 41081.1 | 330.4 | 28307.4 | 89.7 |
| 63 | 96-48-0 | oxolan-2-one | 20000 ^m^ | 5.6 | 0.0 | 0.0 | 0.0 | 16.2 | 0.5 |
| 64 | 110-38-3 | ethyl decanoate | 1120 ^a^ | 389.6 | 1.2 | 154.5 | 0.3 | 484.8 | 2.8 |
| 65 | 623-21-2 | furan-2-ylmethyl butanoate | 4900 ^d^ | 99.1 | 0.9 | 63.1 | 1.3 | 84.0 | 1.4 |
| 66 | 122-78-1 | 2-phenylacetaldehyde | 262 ^d^ | 3441.0 | 13.8 | 3548.2 | 63.6 | 5004.4 | 19.8 |
| 67 | 93-89-0 | ethyl benzoate | 407 ^a^ | 212.0 | 0.8 | 433.5 | 1.0 | 71.1 | 1.1 |
| 68 | 98-86-2 | 1-phenylethanone | 256 ^a^ | 67.9 | 1.2 | 87.9 | 1.2 | 100.9 | 1.9 |
| 69 | 143-08-8 | nonan-1-ol | 806 ^d^ | 59.9 | 3.9 | 37.6 | 0.9 | 133.4 | 1.4 |
| 70 | 503-74-2 | 3-methylbutanoic acid | 1050 ^a^ | 53940.1 | 21.5 | 21089.8 | 26.7 | 15960.2 | 12.7 |
| 71 | 123-25-1 | diethyl butanedioate | 353193 ^a^ | 2189.0 | 17.9 | 1585.7 | 22.5 | 8276.8 | 9.3 |
| 72 | 695-06-7 | 5-ethyloxolan-2-one | 12500 ^d^ | 224.0 | 10.5 | 219.8 | 1.4 | 260.6 | 1.4 |
| 73 | 109-52-4 | pentanoic acid | 389 ^a^ | 19069.7 | 5.3 | 16293.6 | 19.9 | 7275.6 | 1.8 |
| 74 | 627-90-7 | ethyl undecanoate | 1000 ^l^ | 6.5 | 0.0 | 7.1 | 0.0 | 9.6 | 0.0 |
| 75 | 101-97-3 | ethyl 2-phenylacetate | 407 ^a^ | 435.8 | 9.3 | 364.2 | 6.4 | 888.5 | 5.1 |
| 76 | 105-21-5 | 5-propyloxolan-2-one | 1000 ^a^ | 5.6 | 0.1 | 0.0 | 0.0 | 0.0 | 0.0 |
| 77 | 23726-93-4 | β-damascenone | 0.12 ^c^ | 6.6 | 0.1 | 16.5 | 0.1 | 22.9 | 0.5 |
| 78 | 103-45-7 | 2-phenylethyl acetate | 909 ^a^ | 365.1 | 5.3 | 314.0 | 3.7 | 223.9 | 0.2 |
| 79 | 142-62-1 | hexanoic acid | 2520 ^a^ | 359764.4 | 165.5 | 142031.1 | 273.3 | 116599.4 | 142.3 |
| 80 | 106-33-2 | ethyl dodecanoate | 400 ^g^ | 1995.1 | 11.7 | 2354.9 | 25.1 | 6039.9 | 19.6 |
| 81 | 100-51-6 | phenylmethanol | 40927 ^a^ | 24.2 | 0.8 | 19.4 | 0.2 | 13.8 | 0.3 |
| 82 | 2021-28-5 | ethyl 3-phenylpropanoate | 125 ^a^ | 383.8 | 2.8 | 552.3 | 3.0 | 110.1 | 1.4 |
| 83 | 104-50-7 | 5-butyloxolan-2-one | 2816 ^a^ | 6.5 | 0.1 | 7.5 | 0.1 | 10.0 | 0.3 |
| 84 | 60-12-8 | 2-phenylethanol | 28900 ^a^ | 820.2 | 10.8 | 775.1 | 13.2 | 202.0 | 7.8 |
| 85 | 93-51-6 | 2-methoxy-4-methylphenol | 315 ^a^ | 149.2 | 1.1 | 106.8 | 2.3 | 0.0 | 0.0 |
| 86 | 111-14-8 | heptanoic acid | 13281 ^a^ | 2848.9 | 6.9 | 6041.5 | 12.3 | 2497.9 | 3.0 |
| 87 | 104-61-0 | 5-pentyloxolan-2-one | 90.7 ^a^ | 84.6 | 0.9 | 58.6 | 0.9 | 83.6 | 0.4 |
| 88 | 2785-89-9 | 4-ethyl-2-methoxyphenol | 123 ^a^ | 137.4 | 0.2 | 16.6 | 0.2 | 12.0 | 0.5 |
| 89 | 124-06-1 | ethyl tetradecanoate | 33551 ^b^ | 750.3 | 1.6 | 724.1 | 6.7 | 1041.7 | 4.9 |
| 90 | 124-07-2 | octanoic acid | 2700 ^a^ | 4018.5 | 6.9 | 2182.7 | 6.5 | 2671.2 | 11.2 |
| 91 | 106-44-5 | 4-methylphenol | 167 ^a^ | 485.7 | 6.4 | 0.0 | 0.0 | 402.7 | 2.0 |
| 92 | 112-05-0 | nonanoic acid | 3559 ^a^ | 327.2 | 7.5 | 385.4 | 1.2 | 112.6 | 0.6 |
| 93 | 7786-61-0 | 4-ethenyl-2-methoxyphenol | 209 ^a^ | 75.9 | 0.5 | 51.3 | 1.7 | 72.2 | 0.8 |
| 94 | 123-07-9 | 4-ethylphenol | 123 ^a^ | 11.9 | 0.1 | 7.5 | 0.1 | 2.2 | 0.0 |
| 95 | 628-97-7 | ethyl hexadecanoate | 2000 ^a^ | 450.1 | 0.5 | 460.5 | 8.0 | 582.8 | 0.5 |
| 96 | 121-33-5 | 4-hydroxy-3-methoxybenzaldehyde | 439 ^a^ | 34.0 | 0.0 | 40.3 | 0.5 | 0.0 | 0.0 |

Note: ^a^ from reference (W. Fan & Xu, 2011); ^b^ from reference (Ma et al., 2025); ^c^ from reference (Pineau et al., 2007); ^d^ from reference (Feng et al., 2025); ^e^ from reference (Lin, Fan, Xu, Zhu, Yang, & Li, 2024); ^f^ from reference (Gao, Fan, & Xu, 2014); ^g^ from reference (P. P. Chen et al., 2022); ^h^ from reference (Zhou, 2019); ^i^ from reference (H. Y. Fan, Fan, & Xu, 2015); ^l^ from reference (Tan & Siebert, 2004); ^m^ from reference (Peinado, Mauricio, Medina, & Moreno, 2004); ^n^ from reference (T. Zhao et al., 2019); ^p^ from reference (Dong, 2020)

**Supplementary Table 3 (continued) Quantitative data of odor-active compounds (52% vol)**

| No. | CAS | Compounds | Threshold (μg/L) | Concentration (μg/L) | | | | | | | |
| --- | --- | --- | --- | --- | --- | --- | --- | --- | --- | --- | --- |
|  |  |  |  | S-Y1-G-Mean | S-Y1-G-SD | S-Y2-G-Mean | S-Y2-G-SD | S-Y3-G-Mean | S-Y3-G-SD | S-Y4-G-Mean | S-Y4-G-SD |
| 1 | 105-57-7 | 1,1-diethoxyethane | 2090 ^a^ | 134601.4 | 94.8 | 136656.6 | 13.0 | 146907.7 | 72.8 | 123372.2 | 318.3 |
| 2 | 141-78-6 | ethyl acetate | 32552 ^a^ | 936262.0 | 206.9 | 1002593.7 | 88.3 | 1047506.2 | 818.5 | 805475.6 | 39.4 |
| 3 | 97-62-1 | ethyl 2-methylpropanoate | 57.5 ^a^ | 6813.0 | 47.1 | 4476.5 | 0.9 | 5723.8 | 5.4 | 7338.8 | 7.4 |
| 4 | 110-62-3 | pentanal | 725 ^d^ | 6319.8 | 22.9 | 6438.7 | 9.9 | 4216.8 | 34.1 | 5250.3 | 26.9 |
| 5 | 78-92-2 | butan-2-ol | 50000 ^m^ | 88840.3 | 520.0 | 135349.5 | 221.4 | 99490.6 | 463.2 | 97421.5 | 213.2 |
| 6 | 110-19-0 | 2-methylpropyl acetate | 922 ^a^ | 3381.1 | 30.2 | 3178.2 | 21.8 | 2264.5 | 17.8 | 5734.7 | 13.5 |
| 7 | 105-54-4 | ethyl butanoate | 81.5 ^a^ | 135068.7 | 266.3 | 155727.9 | 99.6 | 174412.8 | 100.8 | 88332.3 | 146.4 |
| 8 | 7452-79-1 | ethyl 2-methylbutanoate | 18.0 ^a^ | 3232.1 | 21.5 | 2559.7 | 83.0 | 1860.9 | 17.7 | 2225.5 | 27.7 |
| 9 | 108-64-5 | ethyl 3-methylbutanoate | 6.89 ^a^ | 7009.3 | 13.9 | 6915.2 | 39.2 | 8142.5 | 132.4 | 5600.9 | 12.4 |
| 10 | 590-86-3 | 3-methylbutanal | 16.5 ^a^ | 10227.5 | 11.5 | 12969.8 | 34.0 | 11373.0 | 17.8 | 13100.0 | 30.7 |
| 11 | 123-86-4 | butyl acetate | 1800 ^h^ | 5603.5 | 28.1 | 8598.1 | 61.3 | 4728.4 | 115.3 | 5206.8 | 6.2 |
| 12 | 66-25-1 | hexanal | 25.5 ^a^ | 10750.0 | 46.8 | 11429.0 | 44.7 | 9341.0 | 55.8 | 10879.4 | 48.9 |
| 13 | 78-83-1 | 2-methylpropan-1-ol | 28300 ^f^ | 62859.3 | 73.4 | 59862.4 | 14.7 | 60458.2 | 141.0 | 52328.4 | 2.7 |
| 14 | 71-36-3 | butan-1-ol | 2733 ^a^ | 15023.2 | 13.5 | 13799.5 | 17.2 | 14832.7 | 116.7 | 20180.8 | 35.9 |
| 15 | 6032-29-7 | pentan-2-ol | 194313 ^a^ | 50826.6 | 46.5 | 44439.0 | 45.6 | 46067.5 | 368.6 | 56428.4 | 86.5 |
| 16 | 123-92-2 | 3-methylbutyl acetate | 93.9 ^a^ | 1103.1 | 25.0 | 1353.5 | 5.2 | 983.3 | 22.8 | 1442.6 | 3.0 |
| 17 | 539-82-2 | ethyl pentanoate | 26.8 ^a^ | 8115.3 | 35.4 | 8476.4 | 9.5 | 7122.8 | 13.2 | 7030.4 | 1.8 |
| 18 | 123-51-3 | 3-methylbutan-1-ol | 179191 ^a^ | 189121.4 | 668.6 | 108999.1 | 555.6 | 258645.3 | 404.8 | 123138.9 | 365.5 |
| 19 | 123-66-0 | ethyl hexanoate | 55.3 ^a^ | 1624762.0 | 1077.3 | 1759344.0 | 17063.6 | 1789751.4 | 391.0 | 1489059.9 | 122.6 |
| 20 | 109-08-0 | 2-methylpyrazine | 60000 ^a^ | 635.1 | 12.1 | 470.5 | 5.0 | 1209.0 | 6.7 | 511.1 | 2.7 |
| 21 | 142-92-7 | hexyl acetate | 5560 ^d^ | 1896.2 | 1.3 | 1374.7 | 24.3 | 1542.3 | 16.9 | 1050.6 | 3.9 |
| 22 | 106-27-4 | 3-methylbutyl butanoate | 915 ^f^ | 1954.3 | 4.8 | 1781.5 | 6.0 | 1584.6 | 25.3 | 1944.3 | 20.3 |
| 23 | 108-50-9 | 2,6-dimethylpyrazine | 791 ^a^ | 91.2 | 2.2 | 67.2 | 0.7 | 172.7 | 1.0 | 73.0 | 0.4 |
| 24 | 111-13-7 | octan-2-one | 250 ^l^ | 2.2 | 0.6 | 0.0 | 0.0 | 2.2 | 0.2 | 6.9 | 0.4 |
| 25 | 659-70-1 | 3-methylbutyl 3-methylbutanoate | 134 ^b^ | 36.8 | 0.3 | 37.7 | 0.0 | 33.9 | 0.4 | 31.2 | 0.2 |
| 26 | 626-77-7 | propyl hexanoate | 12784 ^a^ | 2015.3 | 38.2 | 3210.8 | 27.9 | 2194.7 | 8.0 | 2109.2 | 6.3 |
| 27 | 124-13-0 | octanal | 39.6 ^a^ | 6254.5 | 28.3 | 5484.9 | 40.7 | 4831.1 | 114.8 | 4760.0 | 32.3 |
| 28 | 543-49-7 | heptan-2-ol | 1431 ^a^ | 42345.8 | 612.2 | 20741.6 | 618.1 | 7189.0 | 94.3 | 11064.2 | 121.4 |
| 29 | 106-30-9 | ethyl heptanoate | 13153 ^a^ | 39500.0 | 39.3 | 44610.8 | 61.3 | 44553.4 | 209.6 | 28948.9 | 12.8 |
| 30 | 105-79-3 | 2-methylpropyl hexanoate | 5350 ^d^ | 114.0 | 0.6 | 102.9 | 2.4 | 212.4 | 6.0 | 119.8 | 2.1 |
| 31 | 111-27-3 | hexan-1-ol | 5370 ^d^ | 23191.8 | 8.0 | 23705.5 | 0.1 | 26472.8 | 2.1 | 26910.9 | 2.0 |
| 32 | 928-97-2 | (E)-hex-3-en-1-ol | 400 ^n^ | 436.0 | 5.3 | 211.3 | 5.3 | 167.1 | 8.7 | 205.2 | 8.6 |
| 33 | 821-55-6 | nonan-2-one | 393 ^d^ | 254.5 | 12.3 | 250.0 | 3.8 | 331.2 | 0.9 | 361.2 | 7.0 |
| 34 | 589-98-0 | octan-3-ol | 483 ^a^ | 122.3 | 3.4 | 130.0 | 3.1 | 30.3 | 0.7 | 140.6 | 1.3 |
| 35 | 13925-03-6 | 2-ethyl-6-methylpyrazine | 40.0 ^d^ | 24.9 | 0.6 | 20.1 | 1.2 | 30.1 | 1.3 | 13.7 | 0.1 |
| 36 | 3658-80-8 | (methyltrisulfanyl)methane | 0.36 ^a^ | 113.5 | 3.8 | 95.6 | 1.3 | 108.9 | 1.6 | 139.6 | 1.6 |
| 37 | 1669-44-9 | (E)-oct-3-en-2-one | 48.2 ^b^ | 30.4 | 2.2 | 0.0 | 0.0 | 0.0 | 0.0 | 0.0 | 0.0 |
| 38 | 13067-27-1 | 2,6-diethylpyrazine | 6.00 ^d^ | 6.3 | 0.6 | 7.8 | 0.2 | 5.9 | 0.3 | 5.6 | 0.3 |
| 39 | 14667-55-1 | 2,3,5-trimethylpyrazine | 730 ^a^ | 12.4 | 0.5 | 35.6 | 2.7 | 34.6 | 0.5 | 36.2 | 0.8 |
| 40 | 124-19-6 | nonanal | 122 ^a^ | 70.9 | 1.8 | 163.8 | 1.8 | 430.4 | 7.9 | 89.4 | 2.7 |
| 41 | 64-19-7 | acetic acid | 160000 ^f^ | 28930.1 | 1.8 | 48211.3 | 7.1 | 52592.8 | 70.4 | 49603.5 | 43.3 |
| 42 | 106-32-1 | ethyl octanoate | 12.9 ^a^ | 21589.1 | 3.5 | 20882.1 | 36.0 | 14968.3 | 119.2 | 13016.1 | 4.0 |
| 43 | 111-70-6 | heptan-1-ol | 26600 ^i^ | 1810.9 | 5.7 | 1431.1 | 16.0 | 2046.2 | 6.4 | 1607.1 | 35.8 |
| 44 | 7779-80-8 | 2-methylpropyl heptanoate | 26264 ^d^ | 8.3 | 0.2 | 14.2 | 0.2 | 8.3 | 0.2 | 10.4 | 0.3 |
| 45 | 2198-61-0 | 3-methylbutyl hexanoate | 1400 ^e^ | 1078.9 | 7.7 | 1625.9 | 33.7 | 988.6 | 18.4 | 921.2 | 8.3 |
| 46 | 13529-27-6 | 2-(diethoxymethyl)furan | 6172 ^f^ | 2393.2 | 3.2 | 6237.2 | 49.7 | 2113.4 | 13.3 | 4185.3 | 14.8 |
| 47 | 585-24-0 | 2-methylpropyl 2-hydroxypropanoate | 4904 ^b^ | 3010.6 | 8.4 | 2926.9 | 14.0 | 3296.6 | 50.8 | 3162.3 | 38.1 |
| 48 | 1124-11-4 | 2,3,5,6-tetramethylpyrazine | 80073 ^a^ | 377.6 | 0.2 | 387.7 | 7.9 | 415.4 | 8.5 | 246.0 | 2.1 |
| 49 | 98-01-1 | furan-2-carbaldehyde | 44000 ^a^ | 384.1 | 1.3 | 864.6 | 5.2 | 672.0 | 5.6 | 390.9 | 7.3 |
| 50 | 540-07-8 | pentyl hexanoate | 13802 ^d^ | 1051.6 | 16.5 | 1593.4 | 14.1 | 965.0 | 10.3 | 890.7 | 10.0 |
| 51 | 112-31-2 | decanal | 70.8 ^e^ | 216.4 | 6.4 | 293.1 | 0.1 | 310.4 | 4.7 | 160.2 | 3.7 |
| 52 | 100-52-7 | benzaldehyde | 4203 ^a^ | 3339.6 | 13.6 | 3644.5 | 1.4 | 3449.2 | 24.2 | 3429.7 | 12.3 |
| 53 | 624-13-5 | propyl octanoate | 662 ^a^ | 60.3 | 1.9 | 45.5 | 2.3 | 93.4 | 1.0 | 194.1 | 4.1 |
| 54 | 123-29-5 | ethyl nonanoate | 3151 ^a^ | 3053.6 | 4.7 | 6757.8 | 23.8 | 9431.4 | 55.8 | 2778.4 | 12.2 |
| 55 | 79-09-4 | propanoic acid | 18200 ^a^ | 7357.3 | 28.7 | 2240.6 | 14.0 | 7699.9 | 19.1 | 2224.5 | 3.9 |
| 56 | 623-17-6 | furan-2-ylmethyl acetate | 26050 ^p^ | 51.6 | 0.5 | 31.9 | 1.2 | 33.2 | 0.8 | 38.8 | 1.0 |
| 57 | 108-29-2 | 5-methyloxolan-2-one | 2200 ^a^ | 0.0 | 0.0 | 0.0 | 0.0 | 0.0 | 0.0 | 0.0 | 0.0 |
| 58 | 111-87-5 | octan-1-ol | 1100 ^d^ | 345.0 | 21.9 | 71.9 | 0.7 | 299.1 | 1.3 | 116.4 | 70.9 |
| 59 | 79-31-2 | 2-methylpropanoic acid | 1580 ^f^ | 3960.5 | 4.0 | 5074.8 | 11.3 | 4100.1 | 23.8 | 3492.0 | 26.7 |
| 60 | 557-48-2 | (2E,6Z)-nona-2,6-dienal | 0.64 ^b^ | 284.7 | 7.2 | 374.1 | 6.8 | 695.9 | 9.4 | 243.8 | 4.4 |
| 61 | 6378-65-0 | hexyl hexanoate | 1891 ^d^ | 5562.7 | 2.6 | 5252.7 | 15.7 | 4737.8 | 13.1 | 4061.3 | 8.1 |
| 62 | 107-92-6 | butanoic acid | 965 ^a^ | 33189.8 | 180.9 | 29228.7 | 155.8 | 32978.2 | 77.3 | 22392.7 | 205.3 |
| 63 | 96-48-0 | oxolan-2-one | 20000 ^m^ | 9.0 | 0.5 | 4.4 | 0.2 | 0.0 | 0.0 | 9.0 | 0.5 |
| 64 | 110-38-3 | ethyl decanoate | 1120 ^a^ | 214.0 | 0.8 | 379.7 | 1.3 | 484.4 | 4.5 | 123.4 | 1.4 |
| 65 | 623-21-2 | furan-2-ylmethyl butanoate | 4900 ^d^ | 68.6 | 0.9 | 81.9 | 0.6 | 3.3 | 0.2 | 77.7 | 1.8 |
| 66 | 122-78-1 | 2-phenylacetaldehyde | 262 ^d^ | 3161.2 | 11.0 | 3430.3 | 21.5 | 2275.3 | 8.1 | 3231.8 | 17.6 |
| 67 | 93-89-0 | ethyl benzoate | 407 ^a^ | 119.5 | 2.9 | 98.7 | 0.6 | 87.2 | 1.7 | 80.4 | 3.5 |
| 68 | 98-86-2 | 1-phenylethanone | 256 ^a^ | 68.3 | 0.5 | 79.0 | 0.6 | 56.5 | 0.5 | 77.2 | 0.9 |
| 69 | 143-08-8 | nonan-1-ol | 806 ^d^ | 95.9 | 1.5 | 53.6 | 1.1 | 117.3 | 2.0 | 86.0 | 0.5 |
| 70 | 503-74-2 | 3-methylbutanoic acid | 1050 ^a^ | 11662.3 | 12.2 | 15128.0 | 30.7 | 9793.1 | 13.0 | 8416.1 | 14.8 |
| 71 | 123-25-1 | diethyl butanedioate | 353193 ^a^ | 4435.1 | 18.1 | 1614.4 | 22.8 | 7372.2 | 33.0 | 1279.8 | 18.4 |
| 72 | 695-06-7 | 5-ethyloxolan-2-one | 12500 ^d^ | 165.1 | 1.9 | 196.0 | 4.4 | 0.0 | 0.0 | 313.4 | 8.5 |
| 73 | 109-52-4 | pentanoic acid | 389 ^a^ | 9261.5 | 6.2 | 10173.6 | 0.3 | 9335.8 | 12.7 | 9249.5 | 23.5 |
| 74 | 627-90-7 | ethyl undecanoate | 1000 ^l^ | 8.9 | 0.3 | 10.4 | 0.1 | 8.2 | 0.3 | 9.5 | 0.2 |
| 75 | 101-97-3 | ethyl 2-phenylacetate | 407 ^a^ | 564.6 | 4.1 | 578.7 | 11.7 | 305.9 | 9.1 | 439.0 | 2.3 |
| 76 | 105-21-5 | 5-propyloxolan-2-one | 1000 ^a^ | 0.0 | 0.0 | 4.8 | 0.1 | 9.6 | 0.4 | 0.0 | 0.0 |
| 77 | 23726-93-4 | β-damascenone | 0.12 ^c^ | 6.5 | 0.1 | 7.6 | 0.1 | 16.4 | 0.4 | 6.7 | 0.1 |
| 78 | 103-45-7 | 2-phenylethyl acetate | 909 ^a^ | 740.9 | 8.0 | 268.3 | 0.4 | 240.6 | 0.5 | 323.5 | 0.9 |
| 79 | 142-62-1 | hexanoic acid | 2520 ^a^ | 68322.0 | 28.7 | 121980.3 | 54.7 | 133895.6 | 35.5 | 96413.6 | 10.8 |
| 80 | 106-33-2 | ethyl dodecanoate | 400 ^g^ | 1612.3 | 29.9 | 5430.3 | 15.0 | 851.5 | 22.3 | 5572.7 | 12.3 |
| 81 | 100-51-6 | phenylmethanol | 40927 ^a^ | 13.0 | 0.4 | 11.7 | 0.2 | 12.3 | 0.2 | 18.3 | 0.4 |
| 82 | 2021-28-5 | ethyl 3-phenylpropanoate | 125 ^a^ | 214.1 | 4.0 | 532.7 | 12.3 | 134.5 | 2.5 | 159.3 | 1.1 |
| 83 | 104-50-7 | 5-butyloxolan-2-one | 2816 ^a^ | 0.0 | 0.0 | 5.6 | 0.0 | 0.0 | 0.0 | 0.0 | 0.0 |
| 84 | 60-12-8 | 2-phenylethanol | 28900 ^a^ | 153.3 | 2.3 | 333.7 | 3.0 | 204.8 | 7.4 | 540.3 | 1.4 |
| 85 | 93-51-6 | 2-methoxy-4-methylphenol | 315 ^a^ | 78.4 | 15.5 | 0.0 | 0.0 | 0.0 | 0.0 | 0.0 | 0.0 |
| 86 | 111-14-8 | heptanoic acid | 13281 ^a^ | 4160.1 | 13.2 | 4109.1 | 8.2 | 3100.8 | 24.5 | 2041.0 | 4.7 |
| 87 | 104-61-0 | 5-pentyloxolan-2-one | 90.7 ^a^ | 58.2 | 1.4 | 63.7 | 0.6 | 0.0 | 0.0 | 46.7 | 1.3 |
| 88 | 2785-89-9 | 4-ethyl-2-methoxyphenol | 123 ^a^ | 9.2 | 0.1 | 35.6 | 0.5 | 45.0 | 0.3 | 33.4 | 0.3 |
| 89 | 124-06-1 | ethyl tetradecanoate | 33551 ^b^ | 868.2 | 0.3 | 963.2 | 1.2 | 856.9 | 2.8 | 744.8 | 1.5 |
| 90 | 124-07-2 | octanoic acid | 2700 ^a^ | 3578.9 | 30.4 | 9696.4 | 6.9 | 9197.2 | 10.4 | 2427.4 | 15.4 |
| 91 | 106-44-5 | 4-methylphenol | 167 ^a^ | 111.2 | 0.3 | 405.9 | 4.5 | 0.0 | 0.0 | 435.4 | 4.5 |
| 92 | 112-05-0 | nonanoic acid | 3559 ^a^ | 320.4 | 0.9 | 203.9 | 2.8 | 113.0 | 0.5 | 117.5 | 2.9 |
| 93 | 7786-61-0 | 4-ethenyl-2-methoxyphenol | 209 ^a^ | 51.5 | 1.5 | 70.3 | 1.8 | 47.1 | 1.0 | 67.7 | 1.3 |
| 94 | 123-07-9 | 4-ethylphenol | 123 ^a^ | 0.0 | 0.0 | 7.8 | 0.2 | 36.3 | 37.2 | 0.0 | 0.0 |
| 95 | 628-97-7 | ethyl hexadecanoate | 2000 ^a^ | 589.6 | 0.8 | 598.0 | 0.7 | 602.9 | 1.3 | 500.0 | 0.7 |
| 96 | 121-33-5 | 4-hydroxy-3-methoxybenzaldehyde | 439 ^a^ | 31.5 | 0.0 | 0.0 | 0.0 | 0.0 | 0.0 | 40.3 | 7.0 |

**Supplementary Table 3 (continued) Quantitative data of odor-active compounds (52% vol)**

| No. | CAS | Compounds | Threshold (μg/L) | Concentration (μg/L) | | | | | |
| --- | --- | --- | --- | --- | --- | --- | --- | --- | --- |
|  |  |  |  | A-G1-Z-Mean | A-G1-Z-SD | G-J1-Z-Mean | G-J1-Z-SD | J-Y1-Z-Mean | J-Y1-Z-SD |
| 1 | 105-57-7 | 1,1-diethoxyethane | 2090 ^a^ | 102109.52 | 61.44 | 78074.26 | 25.53 | 89923.39 | 40.87 |
| 2 | 141-78-6 | ethyl acetate | 32552 ^a^ | 969265.34 | 274.21 | 910503.91 | 26.83 | 838096.73 | 112.57 |
| 3 | 97-62-1 | ethyl 2-methylpropanoate | 57.5 ^a^ | 11651.54 | 125.02 | 11445.77 | 49.32 | 9790.84 | 14.22 |
| 4 | 110-62-3 | pentanal | 725 ^d^ | 6231.45 | 43.06 | 8219.30 | 48.38 | 5124.24 | 49.49 |
| 5 | 78-92-2 | butan-2-ol | 50000 ^m^ | 98685.27 | 151.24 | 13506.46 | 35.63 | 9956.39 | 24.51 |
| 6 | 110-19-0 | 2-methylpropyl acetate | 922 ^a^ | 4170.51 | 17.44 | 5285.01 | 31.18 | 3024.51 | 52.10 |
| 7 | 105-54-4 | ethyl butanoate | 81.5 ^a^ | 12491.61 | 48.44 | 11597.43 | 178.00 | 10289.25 | 52.05 |
| 8 | 7452-79-1 | ethyl 2-methylbutanoate | 18.0 ^a^ | 1425.10 | 41.02 | 1500.18 | 18.08 | 2179.94 | 6.33 |
| 9 | 108-64-5 | ethyl 3-methylbutanoate | 6.89 ^a^ | 4404.73 | 21.94 | 4085.36 | 10.90 | 3363.15 | 49.23 |
| 10 | 590-86-3 | 3-methylbutanal | 16.5 ^a^ | 8454.55 | 20.64 | 8688.52 | 48.87 | 6910.70 | 19.90 |
| 11 | 123-86-4 | butyl acetate | 1800 ^h^ | 2854.33 | 231.19 | 3244.86 | 18.80 | 4579.53 | 107.20 |
| 12 | 66-25-1 | hexanal | 25.5 ^a^ | 8006.96 | 46.15 | 6264.55 | 61.53 | 5203.49 | 47.83 |
| 13 | 78-83-1 | 2-methylpropan-1-ol | 28300 ^f^ | 41758.09 | 135.05 | 22146.12 | 175.62 | 30093.08 | 37.93 |
| 14 | 71-36-3 | butan-1-ol | 2733 ^a^ | 12231.55 | 28.78 | 6072.35 | 9.78 | 6059.90 | 19.51 |
| 15 | 6032-29-7 | pentan-2-ol | 194313 ^a^ | 21690.20 | 31.73 | 11990.83 | 61.95 | 7296.18 | 5.31 |
| 16 | 123-92-2 | 3-methylbutyl acetate | 93.9 ^a^ | 1068.71 | 6.91 | 762.90 | 8.58 | 205.60 | 3.67 |
| 17 | 539-82-2 | ethyl pentanoate | 26.8 ^a^ | 17475.26 | 67.88 | 11532.79 | 110.74 | 11356.51 | 46.59 |
| 18 | 123-51-3 | 3-methylbutan-1-ol | 179191 ^a^ | 76828.20 | 36.00 | 70191.00 | 3.12 | 85760.52 | 1507.90 |
| 19 | 123-66-0 | ethyl hexanoate | 55.3 ^a^ | 1071907.30 | 216.88 | 928891.96 | 290.09 | 972703.26 | 692.48 |
| 20 | 109-08-0 | 2-methylpyrazine | 60000 ^a^ | 510.93 | 15.10 | 891.76 | 8.95 | 628.98 | 4.72 |
| 21 | 142-92-7 | hexyl acetate | 5560 ^d^ | 226.14 | 1.69 | 94.00 | 0.69 | 549.17 | 8.96 |
| 22 | 106-27-4 | 3-methylbutyl butanoate | 915 ^f^ | 1404.44 | 3.95 | 885.97 | 2.28 | 836.19 | 4.52 |
| 23 | 108-50-9 | 2,6-dimethylpyrazine | 791 ^a^ | 57.90 | 0.86 | 108.91 | 2.67 | 74.39 | 1.05 |
| 24 | 111-13-7 | octan-2-one | 250 ^l^ | 15.63 | 9.79 | 0.00 | 0.00 | 3.82 | 0.01 |
| 25 | 659-70-1 | 3-methylbutyl 3-methylbutanoate | 134 ^b^ | 25.92 | 0.13 | 27.11 | 0.04 | 23.25 | 0.24 |
| 26 | 626-77-7 | propyl hexanoate | 12784 ^a^ | 5192.11 | 44.77 | 6167.07 | 46.93 | 4175.38 | 15.16 |
| 27 | 124-13-0 | octanal | 39.6 ^a^ | 4198.94 | 41.83 | 15577.14 | 59.21 | 3124.33 | 19.75 |
| 28 | 543-49-7 | heptan-2-ol | 1431 ^a^ | 11519.01 | 19.82 | 8335.03 | 48.69 | 6546.33 | 8.68 |
| 29 | 106-30-9 | ethyl heptanoate | 13153 ^a^ | 16333.42 | 68.18 | 11362.44 | 17.93 | 9166.42 | 8.24 |
| 30 | 105-79-3 | 2-methylpropyl hexanoate | 5350 ^d^ | 82.96 | 0.15 | 82.08 | 0.40 | 59.13 | 1.39 |
| 31 | 111-27-3 | hexan-1-ol | 5370 ^d^ | 18198.74 | 0.52 | 19353.91 | 3.14 | 18194.75 | 2.86 |
| 32 | 928-97-2 | (E)-hex-3-en-1-ol | 400 ^n^ | 141.01 | 2.51 | 101.81 | 1.60 | 69.70 | 0.86 |
| 33 | 821-55-6 | nonan-2-one | 393 ^d^ | 444.34 | 4.06 | 189.85 | 2.58 | 153.99 | 2.06 |
| 34 | 589-98-0 | octan-3-ol | 483 ^a^ | 289.81 | 3.98 | 261.31 | 10.38 | 136.57 | 0.76 |
| 35 | 13925-03-6 | 2-ethyl-6-methylpyrazine | 40.0 ^d^ | 5.83 | 0.06 | 10.75 | 0.09 | 7.59 | 0.13 |
| 36 | 3658-80-8 | (methyltrisulfanyl)methane | 0.36 ^a^ | 80.90 | 1.74 | 61.86 | 0.76 | 35.67 | 1.51 |
| 37 | 1669-44-9 | (E)-oct-3-en-2-one | 48.2 ^b^ | 22.99 | 0.20 | 0.00 | 0.00 | 203.11 | 13.44 |
| 38 | 13067-27-1 | 2,6-diethylpyrazine | 6.00 ^d^ | 3.02 | 0.07 | 7.71 | 0.07 | 3.57 | 0.24 |
| 39 | 14667-55-1 | 2,3,5-trimethylpyrazine | 730 ^a^ | 7.51 | 0.05 | 4.07 | 0.09 | 4.51 | 0.12 |
| 40 | 124-19-6 | nonanal | 122 ^a^ | 1316.77 | 23.87 | 1370.34 | 5.68 | 853.56 | 3.19 |
| 41 | 64-19-7 | acetic acid | 160000 ^f^ | 158055.00 | 30.70 | 114440.09 | 36.99 | 104446.55 | 108.18 |
| 42 | 106-32-1 | ethyl octanoate | 12.9 ^a^ | 5720.12 | 16.92 | 4975.88 | 24.93 | 2552.50 | 21.65 |
| 43 | 111-70-6 | heptan-1-ol | 26600 ^i^ | 1899.62 | 15.43 | 721.82 | 0.01 | 1361.18 | 10.73 |
| 44 | 7779-80-8 | 2-methylpropyl heptanoate | 26264 ^d^ | 7.35 | 0.09 | 9.04 | 0.15 | 4.81 | 0.15 |
| 45 | 2198-61-0 | 3-methylbutyl hexanoate | 1400 ^e^ | 532.55 | 4.78 | 494.63 | 1.12 | 211.73 | 2.30 |
| 46 | 13529-27-6 | 2-(diethoxymethyl)furan | 6172 ^f^ | 6122.27 | 39.54 | 6295.55 | 10.57 | 4171.85 | 62.11 |
| 47 | 585-24-0 | 2-methylpropyl 2-hydroxypropanoate | 4904 ^b^ | 1968.50 | 9.84 | 1981.31 | 18.83 | 1664.95 | 5.39 |
| 48 | 1124-11-4 | 2,3,5,6-tetramethylpyrazine | 80073 ^a^ | 109.87 | 0.79 | 602.80 | 2.87 | 264.75 | 4.21 |
| 49 | 98-01-1 | furan-2-carbaldehyde | 44000 ^a^ | 235.30 | 2.90 | 154.77 | 2.22 | 121.61 | 0.74 |
| 50 | 540-07-8 | pentyl hexanoate | 13802 ^d^ | 896.90 | 7.19 | 1004.83 | 6.27 | 480.33 | 18.53 |
| 51 | 112-31-2 | decanal | 70.8 ^e^ | 742.13 | 6.58 | 897.64 | 0.45 | 544.16 | 15.31 |
| 52 | 100-52-7 | benzaldehyde | 4203 ^a^ | 1864.85 | 23.25 | 2028.54 | 17.68 | 9363.39 | 41.05 |
| 53 | 624-13-5 | propyl octanoate | 662 ^a^ | 0.00 | 0.00 | 110.20 | 2.71 | 0.00 | 0.00 |
| 54 | 123-29-5 | ethyl nonanoate | 3151 ^a^ | 5828.64 | 29.64 | 2218.47 | 13.31 | 9457.17 | 226.46 |
| 55 | 79-09-4 | propanoic acid | 18200 ^a^ | 13339.41 | 46.54 | 10985.05 | 12.05 | 8544.38 | 12.10 |
| 56 | 623-17-6 | furan-2-ylmethyl acetate | 26050 ^p^ | 36.75 | 0.62 | 53.98 | 1.83 | 33.79 | 1.74 |
| 57 | 108-29-2 | 5-methyloxolan-2-one | 2200 ^a^ | 0.00 | 0.00 | 0.00 | 0.00 | 5.96 | 0.43 |
| 58 | 111-87-5 | octan-1-ol | 1100 ^d^ | 186.26 | 0.73 | 173.12 | 1.57 | 186.40 | 0.54 |
| 59 | 79-31-2 | 2-methylpropanoic acid | 1580 ^f^ | 18877.33 | 17.81 | 14669.10 | 25.17 | 9055.48 | 17.88 |
| 60 | 557-48-2 | (2E,6Z)-nona-2,6-dienal | 0.64 ^b^ | 1463.12 | 18.77 | 1390.59 | 8.95 | 1572.44 | 12.11 |
| 61 | 6378-65-0 | hexyl hexanoate | 1891 ^d^ | 2216.10 | 6.28 | 2357.61 | 10.59 | 3564.07 | 15.76 |
| 62 | 107-92-6 | butanoic acid | 965 ^a^ | 58159.00 | 379.78 | 55162.34 | 195.28 | 48650.61 | 146.68 |
| 63 | 96-48-0 | oxolan-2-one | 20000 ^m^ | 6.85 | 0.33 | 19.66 | 0.43 | 23.19 | 0.68 |
| 64 | 110-38-3 | ethyl decanoate | 1120 ^a^ | 53.25 | 0.52 | 118.25 | 0.46 | 205.25 | 0.44 |
| 65 | 623-21-2 | furan-2-ylmethyl butanoate | 4900 ^d^ | 36.72 | 0.83 | 61.76 | 0.86 | 84.56 | 0.72 |
| 66 | 122-78-1 | 2-phenylacetaldehyde | 262 ^d^ | 1077.62 | 2.96 | 1112.73 | 23.50 | 100.66 | 1.73 |
| 67 | 93-89-0 | ethyl benzoate | 407 ^a^ | 67.62 | 0.44 | 122.92 | 5.16 | 38.01 | 0.40 |
| 68 | 98-86-2 | 1-phenylethanone | 256 ^a^ | 411.36 | 7.36 | 215.32 | 2.89 | 114.85 | 1.50 |
| 69 | 143-08-8 | nonan-1-ol | 806 ^d^ | 1982.60 | 10.04 | 1382.93 | 19.66 | 1547.37 | 8.27 |
| 70 | 503-74-2 | 3-methylbutanoic acid | 1050 ^a^ | 31196.14 | 66.09 | 21758.70 | 36.68 | 22989.83 | 11.87 |
| 71 | 123-25-1 | diethyl butanedioate | 353193 ^a^ | 1891.33 | 14.33 | 1611.29 | 9.47 | 6311.54 | 139.54 |
| 72 | 695-06-7 | 5-ethyloxolan-2-one | 12500 ^d^ | 5.57 | 0.02 | 8.05 | 0.31 | 6.55 | 0.21 |
| 73 | 109-52-4 | pentanoic acid | 389 ^a^ | 14501.17 | 4.65 | 15863.10 | 18.24 | 10238.82 | 3.58 |
| 74 | 627-90-7 | ethyl undecanoate | 1000 ^l^ | 5.49 | 0.15 | 6.52 | 0.10 | 6.32 | 1.03 |
| 75 | 101-97-3 | ethyl 2-phenylacetate | 407 ^a^ | 347.54 | 11.06 | 334.45 | 1.39 | 1334.83 | 15.19 |
| 76 | 105-21-5 | 5-propyloxolan-2-one | 1000 ^a^ | 3.10 | 0.08 | 3.56 | 0.05 | 5.82 | 0.25 |
| 77 | 23726-93-4 | β-damascenone | 0.12 ^c^ | 0.00 | 0.00 | 0.00 | 0.00 | 0.00 | 0.00 |
| 78 | 103-45-7 | 2-phenylethyl acetate | 909 ^a^ | 218.91 | 0.29 | 216.84 | 0.25 | 232.23 | 0.58 |
| 79 | 142-62-1 | hexanoic acid | 2520 ^a^ | 212807.63 | 144.02 | 244974.24 | 373.47 | 222207.39 | 38.14 |
| 80 | 106-33-2 | ethyl dodecanoate | 400 ^g^ | 1638.51 | 20.52 | 2333.24 | 12.10 | 4220.34 | 206.23 |
| 81 | 100-51-6 | phenylmethanol | 40927 ^a^ | 41.29 | 8.67 | 30.47 | 0.81 | 26.44 | 0.50 |
| 82 | 2021-28-5 | ethyl 3-phenylpropanoate | 125 ^a^ | 626.88 | 7.63 | 532.60 | 6.18 | 192.91 | 0.36 |
| 83 | 104-50-7 | 5-butyloxolan-2-one | 2816 ^a^ | 4.47 | 0.08 | 5.58 | 0.02 | 6.51 | 0.19 |
| 84 | 60-12-8 | 2-phenylethanol | 28900 ^a^ | 810.99 | 3.54 | 0.00 | 0.00 | 64.40 | 8.52 |
| 85 | 93-51-6 | 2-methoxy-4-methylphenol | 315 ^a^ | 0.00 | 0.00 | 84.12 | 0.87 | 0.00 | 0.00 |
| 86 | 111-14-8 | heptanoic acid | 13281 ^a^ | 4222.05 | 1.53 | 4300.53 | 11.63 | 3564.61 | 1.93 |
| 87 | 104-61-0 | 5-pentyloxolan-2-one | 90.7 ^a^ | 65.21 | 0.37 | 73.59 | 1.27 | 53.49 | 0.86 |
| 88 | 2785-89-9 | 4-ethyl-2-methoxyphenol | 123 ^a^ | 28.14 | 0.48 | 85.84 | 0.54 | 61.52 | 0.65 |
| 89 | 124-06-1 | ethyl tetradecanoate | 33551 ^b^ | 617.10 | 1.72 | 394.40 | 5.44 | 475.51 | 1.03 |
| 90 | 124-07-2 | octanoic acid | 2700 ^a^ | 2166.94 | 9.24 | 1827.12 | 9.85 | 1944.19 | 1.91 |
| 91 | 106-44-5 | 4-methylphenol | 167 ^a^ | 396.67 | 10.58 | 19.21 | 0.00 | 464.65 | 1.62 |
| 92 | 112-05-0 | nonanoic acid | 3559 ^a^ | 332.47 | 1.27 | 114.68 | 1.70 | 352.99 | 3.37 |
| 93 | 7786-61-0 | 4-ethenyl-2-methoxyphenol | 209 ^a^ | 1.95 | 0.09 | 3.33 | 0.13 | 0.00 | 0.00 |
| 94 | 123-07-9 | 4-ethylphenol | 123 ^a^ | 10.32 | 0.31 | 4.76 | 0.07 | 6.16 | 0.13 |
| 95 | 628-97-7 | ethyl hexadecanoate | 2000 ^a^ | 324.39 | 0.37 | 328.07 | 7.21 | 320.34 | 3.84 |
| 96 | 121-33-5 | 4-hydroxy-3-methoxybenzaldehyde | 439 ^a^ | 37.03 | 0.21 | 30.58 | 0.00 | 0.00 | 0.00 |

**Supplementary Table 3 (continued) Quantitative data of odor-active compounds (52% vol)**

| No. | CAS | Compounds | Threshold (μg/L) | Concentration (μg/L) | | | | | | | |
| --- | --- | --- | --- | --- | --- | --- | --- | --- | --- | --- | --- |
|  |  |  |  | S-Y1-Z-Mean | S-Y1-Z-SD | S-Y2-Z-Mean | S-Y2-Z-SD | S-Y3-Z-Mean | S-Y3-Z-SD | S-Y4-Z-Mean | S-Y4-Z-SD |
| 1 | 105-57-7 | 1,1-diethoxyethane | 2090 ^a^ | 111146.84 | 66.50 | 116199.53 | 17.19 | 93821.22 | 144.38 | 86882.03 | 158.45 |
| 2 | 141-78-6 | ethyl acetate | 32552 ^a^ | 984087.72 | 719.84 | 806177.21 | 50.40 | 892777.04 | 172.83 | 703756.21 | 47.30 |
| 3 | 97-62-1 | ethyl 2-methylpropanoate | 57.5 ^a^ | 14561.43 | 47.99 | 16624.97 | 44.64 | 12690.32 | 66.54 | 13327.97 | 48.84 |
| 4 | 110-62-3 | pentanal | 725 ^d^ | 6262.92 | 28.41 | 6210.60 | 49.59 | 6063.77 | 3.02 | 9393.58 | 31.88 |
| 5 | 78-92-2 | butan-2-ol | 50000 ^m^ | 87374.32 | 91.66 | 83031.53 | 486.07 | 82238.85 | 451.23 | 134081.35 | 604.14 |
| 6 | 110-19-0 | 2-methylpropyl acetate | 922 ^a^ | 1918.33 | 3.62 | 2119.22 | 2.39 | 1524.27 | 20.27 | 1482.35 | 6.08 |
| 7 | 105-54-4 | ethyl butanoate | 81.5 ^a^ | 52478.61 | 24.97 | 28431.66 | 105.76 | 55316.83 | 59.23 | 27723.80 | 140.84 |
| 8 | 7452-79-1 | ethyl 2-methylbutanoate | 18.0 ^a^ | 1666.03 | 9.13 | 1354.86 | 3.15 | 1526.65 | 26.88 | 1543.62 | 14.12 |
| 9 | 108-64-5 | ethyl 3-methylbutanoate | 6.89 ^a^ | 6003.75 | 14.56 | 4761.42 | 39.82 | 7536.53 | 10.56 | 4346.23 | 30.65 |
| 10 | 590-86-3 | 3-methylbutanal | 16.5 ^a^ | 8798.91 | 53.93 | 7852.69 | 32.33 | 7633.08 | 17.90 | 9545.82 | 56.68 |
| 11 | 123-86-4 | butyl acetate | 1800 ^h^ | 2899.44 | 19.66 | 4150.31 | 37.86 | 2740.00 | 36.28 | 3641.31 | 23.85 |
| 12 | 66-25-1 | hexanal | 25.5 ^a^ | 5251.37 | 11.19 | 6109.62 | 47.94 | 9333.32 | 39.05 | 9917.91 | 39.13 |
| 13 | 78-83-1 | 2-methylpropan-1-ol | 28300 ^f^ | 35357.31 | 9.27 | 38252.03 | 91.86 | 30314.49 | 142.06 | 25845.12 | 179.55 |
| 14 | 71-36-3 | butan-1-ol | 2733 ^a^ | 13040.26 | 67.82 | 9743.16 | 62.08 | 9308.57 | 13.04 | 14150.59 | 10.61 |
| 15 | 6032-29-7 | pentan-2-ol | 194313 ^a^ | 14962.20 | 15.29 | 14934.68 | 30.16 | 16047.39 | 61.17 | 22833.52 | 16.44 |
| 16 | 123-92-2 | 3-methylbutyl acetate | 93.9 ^a^ | 321.24 | 3.16 | 296.98 | 3.76 | 266.04 | 5.99 | 300.61 | 7.84 |
| 17 | 539-82-2 | ethyl pentanoate | 26.8 ^a^ | 14850.97 | 26.36 | 15277.28 | 49.53 | 23756.06 | 48.57 | 13300.44 | 54.75 |
| 18 | 123-51-3 | 3-methylbutan-1-ol | 179191 ^a^ | 186197.83 | 111.36 | 218144.35 | 881.22 | 191628.99 | 503.67 | 93010.45 | 66.47 |
| 19 | 123-66-0 | ethyl hexanoate | 55.3 ^a^ | 1417862.24 | 117.85 | 1397150.35 | 1079.64 | 1471110.15 | 292.84 | 1357713.59 | 2930.69 |
| 20 | 109-08-0 | 2-methylpyrazine | 60000 ^a^ | 811.12 | 16.59 | 1000.59 | 24.45 | 913.69 | 13.17 | 710.79 | 9.97 |
| 21 | 142-92-7 | hexyl acetate | 5560 ^d^ | 524.54 | 9.00 | 2584.64 | 40.15 | 564.07 | 15.87 | 1161.45 | 24.71 |
| 22 | 106-27-4 | 3-methylbutyl butanoate | 915 ^f^ | 1717.45 | 1.36 | 2145.15 | 19.71 | 1409.32 | 11.61 | 1658.26 | 44.25 |
| 23 | 108-50-9 | 2,6-dimethylpyrazine | 791 ^a^ | 89.62 | 2.32 | 110.43 | 4.39 | 98.96 | 0.55 | 69.65 | 3.07 |
| 24 | 111-13-7 | octan-2-one | 250 ^l^ | 0.00 | 0.00 | 3.63 | 0.64 | 0.00 | 0.00 | 0.00 | 0.00 |
| 25 | 659-70-1 | 3-methylbutyl 3-methylbutanoate | 134 ^b^ | 29.65 | 0.27 | 32.75 | 0.25 | 28.14 | 0.12 | 25.69 | 0.54 |
| 26 | 626-77-7 | propyl hexanoate | 12784 ^a^ | 7288.58 | 23.91 | 6265.55 | 12.07 | 4007.99 | 20.63 | 3902.92 | 19.46 |
| 27 | 124-13-0 | octanal | 39.6 ^a^ | 3907.05 | 6.25 | 3440.14 | 13.87 | 3951.04 | 3.65 | 8008.31 | 4.35 |
| 28 | 543-49-7 | heptan-2-ol | 1431 ^a^ | 7405.94 | 204.21 | 8762.00 | 44.35 | 5503.15 | 10.24 | 9253.90 | 149.95 |
| 29 | 106-30-9 | ethyl heptanoate | 13153 ^a^ | 16919.51 | 33.18 | 30254.41 | 89.63 | 17963.67 | 191.61 | 22904.26 | 23.83 |
| 30 | 105-79-3 | 2-methylpropyl hexanoate | 5350 ^d^ | 51.88 | 1.03 | 59.41 | 0.21 | 55.11 | 0.43 | 40.32 | 0.84 |
| 31 | 111-27-3 | hexan-1-ol | 5370 ^d^ | 18529.21 | 2.70 | 19791.08 | 14.25 | 19674.30 | 4.01 | 20242.09 | 3.89 |
| 32 | 928-97-2 | (E)-hex-3-en-1-ol | 400 ^n^ | 162.56 | 2.56 | 184.90 | 1.60 | 127.82 | 2.65 | 106.71 | 2.60 |
| 33 | 821-55-6 | nonan-2-one | 393 ^d^ | 320.22 | 3.75 | 361.92 | 2.19 | 203.63 | 3.92 | 233.09 | 1.77 |
| 34 | 589-98-0 | octan-3-ol | 483 ^a^ | 193.06 | 1.70 | 258.36 | 10.40 | 157.66 | 4.87 | 172.25 | 3.75 |
| 35 | 13925-03-6 | 2-ethyl-6-methylpyrazine | 40.0 ^d^ | 9.33 | 0.12 | 11.23 | 0.22 | 7.64 | 0.13 | 9.44 | 0.13 |
| 36 | 3658-80-8 | (methyltrisulfanyl)methane | 0.36 ^a^ | 74.18 | 0.86 | 62.82 | 0.86 | 63.12 | 0.28 | 93.49 | 1.44 |
| 37 | 1669-44-9 | (E)-oct-3-en-2-one | 48.2 ^b^ | 51.93 | 5.39 | 0.00 | 0.00 | 42.52 | 5.04 | 0.00 | 0.00 |
| 38 | 13067-27-1 | 2,6-diethylpyrazine | 6.00 ^d^ | 8.09 | 0.22 | 6.88 | 0.25 | 9.15 | 0.22 | 5.14 | 0.05 |
| 39 | 14667-55-1 | 2,3,5-trimethylpyrazine | 730 ^a^ | 6.79 | 0.25 | 10.62 | 0.14 | 15.08 | 0.06 | 7.58 | 0.15 |
| 40 | 124-19-6 | nonanal | 122 ^a^ | 924.89 | 19.15 | 937.41 | 0.97 | 1063.75 | 16.05 | 969.86 | 8.77 |
| 41 | 64-19-7 | acetic acid | 160000 ^f^ | 128623.46 | 51.07 | 101206.63 | 30.65 | 79888.25 | 33.74 | 139501.82 | 7.86 |
| 42 | 106-32-1 | ethyl octanoate | 12.9 ^a^ | 7322.84 | 3.72 | 6138.72 | 21.79 | 8307.95 | 38.97 | 6416.68 | 10.37 |
| 43 | 111-70-6 | heptan-1-ol | 26600 ^i^ | 1224.22 | 23.01 | 1490.79 | 20.44 | 1027.82 | 8.92 | 1278.12 | 14.69 |
| 44 | 7779-80-8 | 2-methylpropyl heptanoate | 26264 ^d^ | 4.83 | 0.13 | 5.60 | 0.13 | 6.54 | 0.04 | 23.57 | 27.24 |
| 45 | 2198-61-0 | 3-methylbutyl hexanoate | 1400 ^e^ | 1074.27 | 9.78 | 631.53 | 0.27 | 820.37 | 5.98 | 582.80 | 26.59 |
| 46 | 13529-27-6 | 2-(diethoxymethyl)furan | 6172 ^f^ | 10783.86 | 31.02 | 8620.26 | 53.47 | 2150.82 | 21.19 | 7285.31 | 67.43 |
| 47 | 585-24-0 | 2-methylpropyl 2-hydroxypropanoate | 4904 ^b^ | 3107.80 | 34.50 | 2396.94 | 0.93 | 2437.69 | 30.50 | 3198.46 | 26.54 |
| 48 | 1124-11-4 | 2,3,5,6-tetramethylpyrazine | 80073 ^a^ | 227.54 | 2.17 | 249.04 | 1.49 | 227.62 | 3.79 | 230.65 | 1.29 |
| 49 | 98-01-1 | furan-2-carbaldehyde | 44000 ^a^ | 235.46 | 1.58 | 309.84 | 3.94 | 309.16 | 0.63 | 310.76 | 1.36 |
| 50 | 540-07-8 | pentyl hexanoate | 13802 ^d^ | 854.05 | 6.86 | 1004.07 | 2.66 | 758.43 | 10.72 | 789.46 | 11.32 |
| 51 | 112-31-2 | decanal | 70.8 ^e^ | 738.00 | 19.48 | 543.59 | 3.61 | 796.10 | 49.33 | 712.48 | 1.69 |
| 52 | 100-52-7 | benzaldehyde | 4203 ^a^ | 2145.27 | 23.60 | 4212.92 | 15.64 | 1918.83 | 5.45 | 3111.72 | 40.42 |
| 53 | 624-13-5 | propyl octanoate | 662 ^a^ | 0.00 | 0.00 | 57.44 | 0.11 | 0.00 | 0.00 | 0.00 | 0.00 |
| 54 | 123-29-5 | ethyl nonanoate | 3151 ^a^ | 12046.49 | 35.68 | 8758.20 | 73.46 | 11024.44 | 41.94 | 7259.06 | 82.32 |
| 55 | 79-09-4 | propanoic acid | 18200 ^a^ | 6570.97 | 22.48 | 7785.77 | 29.74 | 6224.13 | 27.33 | 5605.82 | 15.25 |
| 56 | 623-17-6 | furan-2-ylmethyl acetate | 26050 ^p^ | 61.93 | 0.61 | 73.47 | 0.30 | 48.57 | 2.05 | 44.24 | 0.86 |
| 57 | 108-29-2 | 5-methyloxolan-2-one | 2200 ^a^ | 0.00 | 0.00 | 3.58 | 0.04 | 0.00 | 0.00 | 0.00 | 0.00 |
| 58 | 111-87-5 | octan-1-ol | 1100 ^d^ | 321.57 | 3.70 | 199.58 | 2.02 | 415.36 | 2.73 | 213.28 | 4.04 |
| 59 | 79-31-2 | 2-methylpropanoic acid | 1580 ^f^ | 5926.47 | 21.41 | 5335.91 | 22.18 | 7500.71 | 87.33 | 4781.56 | 19.07 |
| 60 | 557-48-2 | (2E,6Z)-nona-2,6-dienal | 0.64 ^b^ | 1185.16 | 38.88 | 832.18 | 6.05 | 1279.93 | 26.04 | 864.83 | 84.79 |
| 61 | 6378-65-0 | hexyl hexanoate | 1891 ^d^ | 4815.21 | 122.94 | 6623.25 | 55.81 | 4109.71 | 6.03 | 5890.44 | 40.70 |
| 62 | 107-92-6 | butanoic acid | 965 ^a^ | 49712.83 | 392.03 | 37799.45 | 295.58 | 30677.37 | 294.16 | 36314.99 | 226.19 |
| 63 | 96-48-0 | oxolan-2-one | 20000 ^m^ | 0.00 | 0.00 | 6.01 | 0.29 | 0.00 | 0.00 | 0.00 | 0.00 |
| 64 | 110-38-3 | ethyl decanoate | 1120 ^a^ | 230.22 | 4.37 | 124.80 | 1.99 | 428.67 | 2.49 | 121.75 | 1.67 |
| 65 | 623-21-2 | furan-2-ylmethyl butanoate | 4900 ^d^ | 41.09 | 0.67 | 48.81 | 0.43 | 32.79 | 0.79 | 22.11 | 0.16 |
| 66 | 122-78-1 | 2-phenylacetaldehyde | 262 ^d^ | 2106.89 | 10.14 | 2102.22 | 9.54 | 2005.89 | 40.38 | 1095.89 | 12.30 |
| 67 | 93-89-0 | ethyl benzoate | 407 ^a^ | 10.67 | 0.31 | 220.91 | 4.29 | 35.12 | 0.63 | 32.55 | 0.72 |
| 68 | 98-86-2 | 1-phenylethanone | 256 ^a^ | 423.43 | 3.10 | 529.51 | 4.64 | 412.51 | 4.42 | 210.45 | 3.05 |
| 69 | 143-08-8 | nonan-1-ol | 806 ^d^ | 3201.04 | 20.87 | 3328.00 | 7.38 | 3024.75 | 22.54 | 2917.09 | 8.50 |
| 70 | 503-74-2 | 3-methylbutanoic acid | 1050 ^a^ | 13062.55 | 29.19 | 13247.22 | 27.39 | 6044.23 | 7.60 | 13909.18 | 21.69 |
| 71 | 123-25-1 | diethyl butanedioate | 353193 ^a^ | 5825.08 | 37.49 | 4784.51 | 63.59 | 5511.56 | 17.40 | 5194.60 | 46.64 |
| 72 | 695-06-7 | 5-ethyloxolan-2-one | 12500 ^d^ | 2.55 | 0.09 | 4.44 | 0.05 | 199.07 | 1.45 | 3.47 | 0.09 |
| 73 | 109-52-4 | pentanoic acid | 389 ^a^ | 10969.33 | 0.78 | 5587.60 | 18.80 | 8515.02 | 12.84 | 10889.07 | 26.94 |
| 74 | 627-90-7 | ethyl undecanoate | 1000 ^l^ | 8.19 | 0.29 | 8.74 | 0.30 | 7.96 | 0.22 | 7.32 | 0.03 |
| 75 | 101-97-3 | ethyl 2-phenylacetate | 407 ^a^ | 1069.36 | 5.88 | 1200.70 | 5.85 | 1234.64 | 18.77 | 969.55 | 6.10 |
| 76 | 105-21-5 | 5-propyloxolan-2-one | 1000 ^a^ | 0.00 | 0.00 | 4.60 | 0.40 | 0.00 | 0.00 | 0.00 | 0.00 |
| 77 | 23726-93-4 | β-damascenone | 0.12 ^c^ | 0.00 | 0.00 | 0.00 | 0.00 | 5.01 | 0.10 | 2.55 | 0.04 |
| 78 | 103-45-7 | 2-phenylethyl acetate | 909 ^a^ | 260.09 | 2.29 | 219.54 | 0.09 | 235.20 | 0.43 | 234.09 | 2.24 |
| 79 | 142-62-1 | hexanoic acid | 2520 ^a^ | 235371.49 | 93.50 | 207275.66 | 188.37 | 198337.02 | 95.60 | 216090.90 | 96.28 |
| 80 | 106-33-2 | ethyl dodecanoate | 400 ^g^ | 3754.80 | 46.71 | 4856.38 | 39.47 | 3683.88 | 35.72 | 3547.30 | 15.96 |
| 81 | 100-51-6 | phenylmethanol | 40927 ^a^ | 25.19 | 0.42 | 34.48 | 2.33 | 25.86 | 2.24 | 25.90 | 0.46 |
| 82 | 2021-28-5 | ethyl 3-phenylpropanoate | 125 ^a^ | 229.14 | 4.78 | 439.12 | 48.33 | 294.26 | 2.64 | 215.03 | 18.30 |
| 83 | 104-50-7 | 5-butyloxolan-2-one | 2816 ^a^ | 6.86 | 0.21 | 7.03 | 0.28 | 0.00 | 0.00 | 0.00 | 0.00 |
| 84 | 60-12-8 | 2-phenylethanol | 28900 ^a^ | 293.82 | 8.09 | 388.97 | 14.31 | 260.42 | 11.72 | 295.69 | 9.19 |
| 85 | 93-51-6 | 2-methoxy-4-methylphenol | 315 ^a^ | 164.69 | 2.83 | 0.00 | 0.00 | 0.00 | 0.00 | 133.33 | 0.61 |
| 86 | 111-14-8 | heptanoic acid | 13281 ^a^ | 2624.50 | 8.11 | 2571.72 | 14.27 | 2948.50 | 9.66 | 5207.34 | 16.81 |
| 87 | 104-61-0 | 5-pentyloxolan-2-one | 90.7 ^a^ | 52.15 | 0.23 | 58.98 | 0.25 | 24.07 | 1.29 | 36.69 | 0.41 |
| 88 | 2785-89-9 | 4-ethyl-2-methoxyphenol | 123 ^a^ | 181.49 | 1.07 | 81.15 | 0.44 | 85.87 | 0.31 | 223.07 | 1.52 |
| 89 | 124-06-1 | ethyl tetradecanoate | 33551 ^b^ | 758.78 | 3.66 | 571.00 | 1.27 | 792.31 | 6.83 | 644.63 | 6.06 |
| 90 | 124-07-2 | octanoic acid | 2700 ^a^ | 4134.35 | 2.03 | 1722.63 | 20.57 | 1656.71 | 21.32 | 2027.87 | 27.08 |
| 91 | 106-44-5 | 4-methylphenol | 167 ^a^ | 881.31 | 3.98 | 19.21 | 0.00 | 857.66 | 12.99 | 19.21 | 0.00 |
| 92 | 112-05-0 | nonanoic acid | 3559 ^a^ | 113.11 | 3.32 | 117.72 | 3.03 | 216.73 | 0.69 | 110.68 | 2.35 |
| 93 | 7786-61-0 | 4-ethenyl-2-methoxyphenol | 209 ^a^ | 2.31 | 0.11 | 2.64 | 0.30 | 0.00 | 0.00 | 0.00 | 0.00 |
| 94 | 123-07-9 | 4-ethylphenol | 123 ^a^ | 1.41 | 0.01 | 2.05 | 0.01 | 13.38 | 0.09 | 2.13 | 0.00 |
| 95 | 628-97-7 | ethyl hexadecanoate | 2000 ^a^ | 312.59 | 0.72 | 305.67 | 0.71 | 301.67 | 1.14 | 387.23 | 0.61 |
| 96 | 121-33-5 | 4-hydroxy-3-methoxybenzaldehyde | 439 ^a^ | 33.27 | 0.08 | 0.00 | 0.00 | 0.00 | 0.00 | 32.91 | 0.22 |
